# Prognostic significance of immune cells in non-small cell lung cancer: meta-analysis

**DOI:** 10.18632/oncotarget.24835

**Published:** 2018-05-15

**Authors:** Ross A. Soo, Zhaojin Chen, Rebecca Siew Yan Teng, Hon-Lyn Tan, Barry Iacopetta, Bee Choo Tai, Richie Soong

**Affiliations:** ^1^ Department of Haematology-Oncology, National University Health System, Singapore; ^2^ Cancer Science Institute of Singapore, National University of Singapore, Singapore; ^3^ Investigational Medicine Unit, National University Health System, Singapore; ^4^ Yong Loo Lin School of Medicine, National University of Singapore, Singapore; ^5^ School of Surgery, The University of Western Australia, Perth, Australia; ^6^ Saw Swee Hock School of Public Health, National University of Singapore, Singapore; ^7^ Department of Pathology, National University Health System, Singapore

**Keywords:** non-small cell lung cancer, immune cells, dendritic cells, tumor associated macrophages, mast cells

## Abstract

**Background:**

Tumor-associated immune cells are prognostic in non-small cell lung cancer (NSCLC) but findings have been conflicting.

**Objectives:**

To determine the prognostic role of immune cells according to localization in NSCLC patients.

**Methods:**

A systematic literature review and meta-analysis was performed on dendritic cell (DC), tumor associated macrophages (TAM), mast cells (MC), natural killer (NK) cells, T and B cells and tumor CTLA-4 and PD-L1 studies.

**Results:**

We analysed 96 articles (*n*= 21,752 patients). Improved outcomes were seen with increased tumor DCs (overall survival (OS) hazard ratio (HR) 0.55; 95% confidence interval (CI) 0.44–0.68), NK cells (OS HR 0.45; 0.31–0.65), TAMs (OS HR 0.33; 0.17–0.62), M1 TAMs (OS HR 0.10; 0.05–0.21), CD3+ T cells (disease specific survival (DSS) HR 0.64; 0.48–0.86), CD8+ T cells (OS HR 0.78; 0.66–0.93), B cells (OS HR 0.65; 0.42–0.99) and with increased stroma DC (DSS HR 0.62; 0.47–0.83), NK cells (DSS HR 0.51; 0.32–0.82), M1 TAMs (OS HR 0.63; 0.42–0.94), CD4+ T cells (OS HR 0.45; 0.21–0.94), CD8+ T cells (OS HR 0.77; 0.69–0.86) and B cells (OS HR 0.74;0.56–0.99). Poor outcomes were seen with stromal M2 TAMs (OS HR 1.44; 1.06–1.96) and Tregs (relapse free survival (RFS) HR 1.80; 1.34–2.43). Tumor PD-L1 was associated with worse OS (1.40; 1.20–1.69), RFS (1.67) and DFS (1.24).

**Conclusion:**

Tumor and stroma DC, NK cells, M1 TAMs, CD8+ T cells and B cells were associated with improved prognosis and tumor PD-L1, stromal M2 TAMs and Treg cells had poorer prognosis. Higher quality studies are required for confirmation.

## INTRODUCTION

Lung cancer is one of the most common malignancies globally, accounting for 1.5 million cases annually. It is also the leading cause of cancer deaths globally, causing 1.3 million deaths annually [1]. The tumor microenvironment has a major role in influencing cancer development [2], of which immune cells are considered to contribute to tumor destruction, as well as tumor development by promoting growth and invasion [3, 4].

In recent times, a major advance in the treatment of non-small cell lung cancer (NSCLC) has been the use of immunotherapy, such as immune checkpoint inhibitors targeting cytotoxic T lymphocyte antigen-4 (CTLA-4), programmed death receptor-1 (PD-1) and programmed death receptor ligand-1 (PD-L1) [5]. In the advanced stage NSCLC setting, many PD-1/PD-L1 inhibitors have been approved for use [6], although results from trials in the resected tumor setting have been less encouraging [7].

The potential of the immune system to contribute functionally to both tumor elimination and promotion, and the observed significant effects of its modulation through immunotherapy, have supported that the immune system can be a significant determinant of the outcomes of NSCLC patients. As such, numerous studies have investigated the prognostic and predictive significance of many different cell types of the immune system over the years [4]. The different immune cell types have included mast cells, dendritic cells, natural killer cells, macrophages, neutrophils of the innate immune system, T and B lymphocytes of the adaptive immune system, as well as CTLA-4 and PD-L1-expressing cells targeted by immunotherapy. In many cases, specific subtypes of immune cells, such as M1 and M2 macrophages, and CD3+, CD4+, CD8+, and regulatory T cells have been examined. Moreover, assessment according to localization of the immune cells in tumor parenchyma or stroma has also been performed. This is based on the observed varied presence of these cells in the tissue compartments, and associated functional implications.

Findings from such reports have been numerous and varied according to immune cell type, outcome endpoint, tissue localization, study quality, as well as results. This study was undertaken with the goal of consolidating knowledge on the prognostic significance of the many immune cell types in NSCLC, and according to investigated co-factors.

## METHODS

### Search strategy

Meta-analysis was conducted according to the guidelines of the Systematic Reviews and Meta-Analyses (PRISMA) [8]. Relevant articles were identified through a systematic search of PUBMED using the MeSH terms: “Immune cell type” AND “lung neoplasm” limited to “Human”, “English”. MeSH terms for the immune cell types were “mast cells”, “macrophage”, “dendritic cell”, “NK cell”, “regulatory T cell”, “CD3 T-lymphocyte”, “CD4 T-lymphocyte”, “CD8 T-lymphocyte”, “B cell”, “CTLA-4 antigen” and “Antigen, CD274” (PD-L1). Articles published up to 7 July 2017 were included in our search.

### Inclusion and exclusion criteria

The inclusion criteria for articles were those that reported on samples from patients with primary lung tumors with NSCLC, having no systemic treatment or radiation therapy prior to sample collection, and sufficient prognostic information to determine pooled Hazard Ratios (HR). Where HRs were not reported, included studies had to have sufficient information to extrapolate HR. The exclusion criteria included studies on blood or other body fluids or pre-clinical models, studies on the optimization of immunohistochemistry (IHC) or quantitative immunoflurorescence (QIF) methods, or using non-IHC/QIF based methods to detect immune cells, as well as letters and case reports. References cited in retrieved articles were checked for additional relevant articles. Data from other reviews and meta-analyses were not included, but articles identified through references cited were reviewed. Irrelevant and/or duplicate studies were removed by manual curation. Study eligibility was assessed independently by two authors (RAS and ZC).

### Data extraction

Two investigators (RAS and ZC) independently extracted the data. The following details were extracted from each study: first author, publication year, PMID, country of origin of the study population, immune cell studied, phenotype, markers used to define immune cell type, localization of immune cells (defined as “tumor”, “stroma” or, if the localization was unspecified, “general” compartment), sample size, number of events, tumor stage, treatment setting, and histology (adenocarcinoma, squamous cell carcinoma or mixed). For study methodology, data was collected on the assay used, tissue sample used (full tissue sections or tissue microarray), antibodies used, scoring method and thresholds used to define expression. Survival outcomes annotated included disease-free survival (DFS), relapse-free survival (RFS), disease-specific survival (DSS) and overall survival (OS).

### Assessment of study quality and risk of bias

RAS and HLT independently assessed study quality according to the criteria developed by McShane 2005 and Hayes [9, 10] for tumor marker prognostic studies. In brief, the criteria assessed seven domains including: inclusion and exclusion criteria, prospective or retrospective study design, patient and tumor characteristics, method or assay description, outcome measures defined, patient follow up and number of patients lost to follow-up or otherwise unavailable for analysis.

### Statistical analysis

The prognostic effect of an immune cell was quantified by HR, defined as the relative hazard of death or disease progression of patients with high or positive immune cell levels against those with low or negative immune cell levels. Where HRs were not reported, they were estimated using hazard ratio, odds ratio, or the ratio of median survival, as proposed by Parmar [11]. Stratified analysis was conducted according to localization (tumor, stroma, general), or phenotype of immune cells. A meta-analysis was performed when there were at least two studies in a stratum. Therefore, a single study with a reported HR in an analytic stratum was not analyzed. For studies with considerable heterogeneity, studies were modelled for random-effects, according to the methods of DerSimonian and Laird [12]. Otherwise, a fixed-effect model was used. Heterogeneity was considered to be low, moderate, and high for I^2^ values of 25–50%, 50–75%, and >75%, respectively [13]. Results for each immune cell type were displayed using a forest plot. A funnel plot was constructed to visualize small-study effects and possible publication bias for a stratum with five or more studies. To test for small-study effect, the Egger’s test was subsequently performed when there were at least 10 studies in a stratum. Median survival times were derived from the Kaplan-Meier survival curves using DigitizeIt 2.2. All analyses were performed using StataSE14 (StataCorp LP, College Station, Texas) by assuming a two-sided statistical test with 5% significance level.

## RESULTS

A systematic search of PubMed and referenced articles resulted in 3,291 records, from which 96 individual studies, assessing 21,752 patients, were eligible for meta-analyses (Supplementary Tables 1–2, Supplementary Figure 1). The majority of studies were from East Asia (61, 64%) and mixed NSCLC histology (60, 63%). IHC was used in 92 (96%) of studies, and full tissue sections were used in 67 (70%). The average study quality score for all studies was 4.7.

### Mast cells

**Figure 1:**
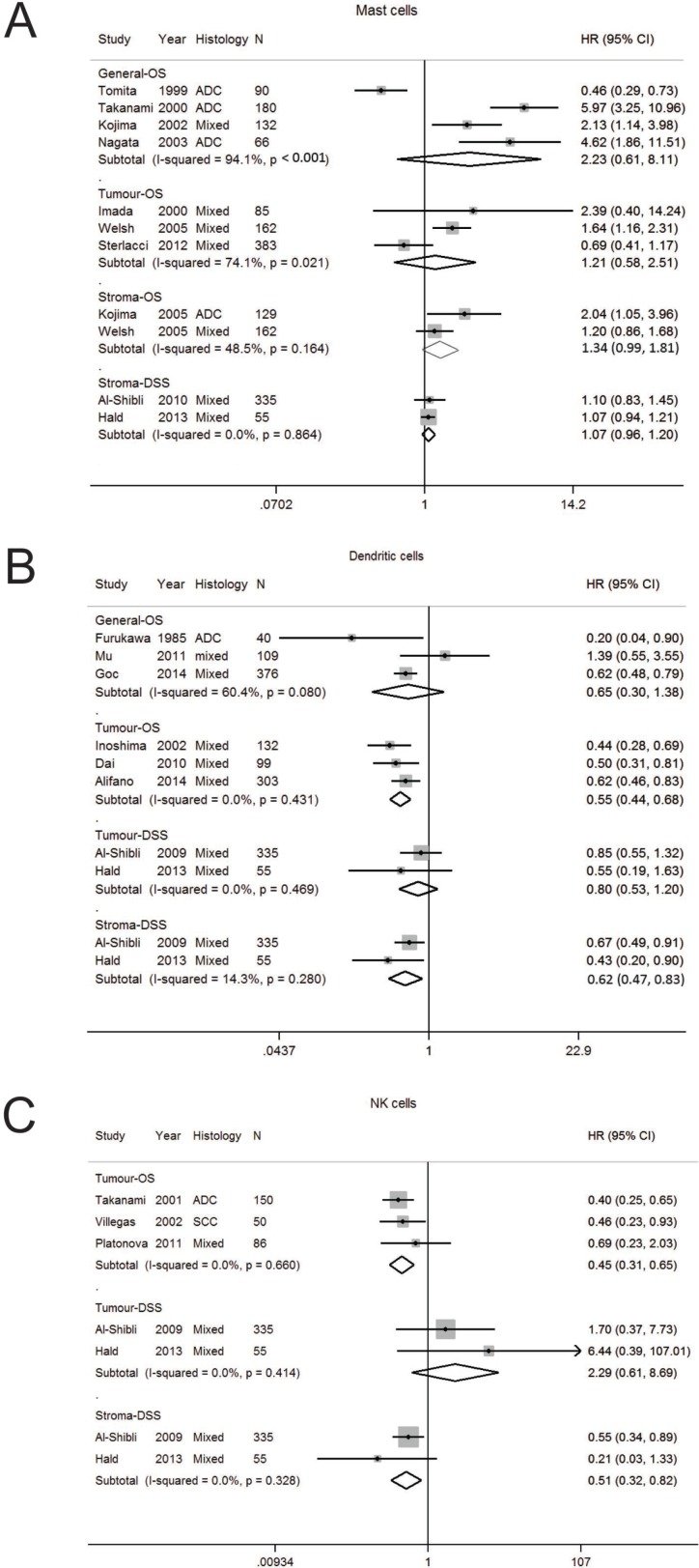
Forest plot of studies assessing (**A**) Mast cells, (**B**) dendritic cells, (**C**) Natural killer cells and survival in patients with non-small cell lung cancer. Adenocarcinoma, ADC; confidence interval, CI; disease specific survival, DSS; hazard ratio, HR; overall survival, OS; programmed cell death-ligand 1, PD-L1; progression free survival, PFS; squamous cell carcinoma, SCC.

Mast cells (MC) play a key role in allergic diseases but are also involved in immune responses. Depending on the type of solid tumor, mast cells can enhance adaptive immunity but also play a key role in tumor angiogenesis, tumor invasion, and immune suppression [14]. Ten studies were analysed [15–24]. (Supplementary Table 1 and 2, Supplementary Figure 1A). The number of studies analysed per stratum ranged from two to three (Table 1). Early studies reported MC counts without consideration of tumor localisation (Supplementary Table 2). Three out of four studies reported increased MC was associated with a worse OS, however the associations did not reach statistical significance in pooled analysis (HR 2.23; 95% CI 0.61–8.11) (Table 1, Figure 1A). Later studies assessed outcomes according to localisation, and reported MC were not significantly associated with OS in the tumor (HR 1.21; 0.58–2.51) or stroma (HR1.34; 0.99–1.81). A high degree of heterogeneity was seen in studies on OS according to general (I^2^ 94.1%, *p* < 0.001) and tumor (I^2^ 74.1%, *p* = 0.021) localisation.

**Table 1:** Summary of hazard ratios, sample sizes and average quality scores from pooled analyses for cell types according to cellular localization

	General		Tumor		Stroma	
	DSS	OS	DSS	OS	DSS	OS
Mast cells	NA	2.23 [0.61–8.11] n468(4) Q4.25	NA	1.21 [0.58–2.51] N630(3) Q5.3	1.07 [0.96–1.20] n390(2) Q5	1.34 [0.99–1.81] (n291(2) Q6.5
Dendritic cells	NA	0.65 [0.30–1.38] n525(3) Q4	0.80 [0.53–1.20] n390(2) Q5.5	0.55 [0.44–0.68] N534(3) Q4.67	0.62 [0.47–0.83] n390(2) Q2	NA
Natural Killer cells	NA	NA	2.29 [0.61–8.69] n390(2) Q5.5	0.45 [0.31–0.65] N374(3) Q2.67	0.51 [0.32–0.82] n390(2) Q5	NA
Macrophages	NA	2.32 [1.38–3.90] n695(5) Q4.2	0.76 [0.50–1.15] n350(2) Q5	0.33 [0.17–0.62] N443(4) Q5	0.79 [0.59–1.06] n350(2) Q5	1.55 [1.01–2.37] n704(5) Q5.4
Macrophages, M1	NA	NA	NA	0.10 [0.05–0.21] N140(2) Q4.5	NA	0.63 [0.42–0.94] n140(2) Q4.5
Macrophages, M2	NA	NA	NA	0.78 [0.35–1.71] N348(3) Q4.67	2.32 [1.66–3.24] n512(2) Q5^7^	1.44 [1.06–1.96] n853(5) Q5
T cells, CD3+	NA	0.72 [0.53–0.97] n848(4) Q4.5	0.64 [0.48–0.86] n350(2) Q5^2,3^	0.88 [0.74–1.05] N420(3) Q3	1.13 [0.38–3.35] n350(2) Q5	0.86 [0.62–1.18] n325(2) Q3
T cells, CD4+	NA	NA	0.86 [0.61–1.21] n350(2) Q5.5^4^	0.74 [0.48–1.15] N678(4) Q4.5	0.23 [0.06–0.95] n350(2) Q5.5	0.45 [0.21–0.94] n358(3) Q5.67
T cells, CD8+	0.70 [0.48–1.02] n554(3) Q4.34^1^	0.80 [0.56–1.15] n1348(6) Q4.5	0.69 [0.50–0.96] n350(2) Q5.5^5,6^	0.78 [0.66–0.93] N2844(9) Q4.55	0.47 [0.36–0.63] n1187(3) Q5.67^9^	0.77 [0.69–0.86] n2157(8) Q5
T cells, regulatory	NA	1.42 [0.78–2.60] n146(2) Q3.5	1.43 [0.69–2.94] n578(2) Q4^7^	1.00 [0.75–1.34] N578(2) Q4	1.80 [1.34–2.43] n678(3) Q4^7,10^	1.43 [0.69–2.94] n750(5) Q4.4
B cells	NA	NA	NA	0.65 [0.42–0.99] N590(2) Q4.5	NA	0.74 [0.56–0.99] n644(2) Q4.5
PD–L1	NA	NA	1.67 [1.22–2.29] n2245(10) Q4.8^7,8^	1.40 [1.20–1.69] N8970(35) Q5	NA	NA

Results are expressed as pooled HR [95% confidence limit], number of patients (number of studies), Q (average quality score).

DFS = disease-free survival, DSS = disease-specific survival, NA = not analyzable, OS = overall survival, RFS = relapse-free survival.

^1^DFS, no DSS available.

^2^RFS HR = 0.73 [0.40–1.32].

^3^DFS HR = 1.01 [0.54–1.88].

^4^RFS HR = 1.11 [0.73–1.68].

^5^DFS HR = 0.58 [0.32–1.04].

^6^RFS HR = 1.22 [0.82–1.83].

^7^RFS, no DSS available.

^8^DFS HR = 1.24 [1.01–1.52].

^9^DFS HR = 1.69 [1.11–2.55].

^10^DFS HR = 1.24 [1.01–1.52].

### Dendritic cells

Dendritic cells (DC) are the most potent antigen presenting cells and regulate the immune system to respond to foreign antigens while avoiding autoimmunity and therefore are important in cancer, generating both immunity and tolerance [25]. Eight studies (were suitable for analysis (Supplementary Tables 1, 2, Supplementary Figure 1B). The average quality score was 4.5 (Supplementary Table 1) [24, 26–32]. On pooled analysis, the HR for OS for general DC was 0.65 (0.30–1.38) (Table 1, Figure 1B). Inoshima et al. first reported high DCs in the tumor compartment was associated with longer OS [27]. In pooled analysis, increased tumor DC was prognostic for OS (HR 0.55; 0.44–0.68) but not for DSS (HR 0.80; 0.53–1.20). In contrast, stromal DC was significantly associated with DSS (HR 0.62; 0.47–0.83). Study heterogeneity was generally low in the studies examined for OS in tumor and DSS in the tumor and stroma. Funnel plot analysis was not performed as there was an inadequate number of publications per stratum.

### Natural killer (NK) cells

Natural killer (NK) cells are the major effector cells of the innate immune system, and have an important role in the immune response against cancer [33]. Only five studies were suitable for pooled analysis (Supplementary Tables 1, 2 Supplementary Figure 1C) [24, 28, 34–36]. Pooled analysis revealed increased tumor NK cells were associated with an improved OS (HR 0.45; 0.31–0.65) but not DSS (HR 2.29; 0.62–8.69), whereas stromal NK cells were associated with better DSS (HR 0.51; 0.32–0.82) (Table 1, Figure 1C). Study heterogeneity was low. As the number of publications per stratum was only 2 or 3, further studies of adequate sample size should be pursued.

### Tumor associated macrophages

Tumor associated macrophages (TAMs) are part of the innate immune system and have an essential function against foreign pathogens [37]. Eighteen studies were analysed (Supplementary Tables 1, 2, Supplementary Figure 1D) [20, 21, 24, 28, 29, 38–50]. The number of studies per stratum was 2–5 (Table 1). Initial studies reported on tumor-associated macrophages (TAM) in the general compartment [39, 40] and subsequently the importance of tumor localization was first recognised by Eeroloa *et al.* who found increased tumoral TAM was associated with an improved OS and DFS [38]. The significance of TAM localization was extended further in a pivotal paper that reported increased tumor TAM had an improved OS whereas stromal TAMs had worse OS [21].

**Figure 2:**
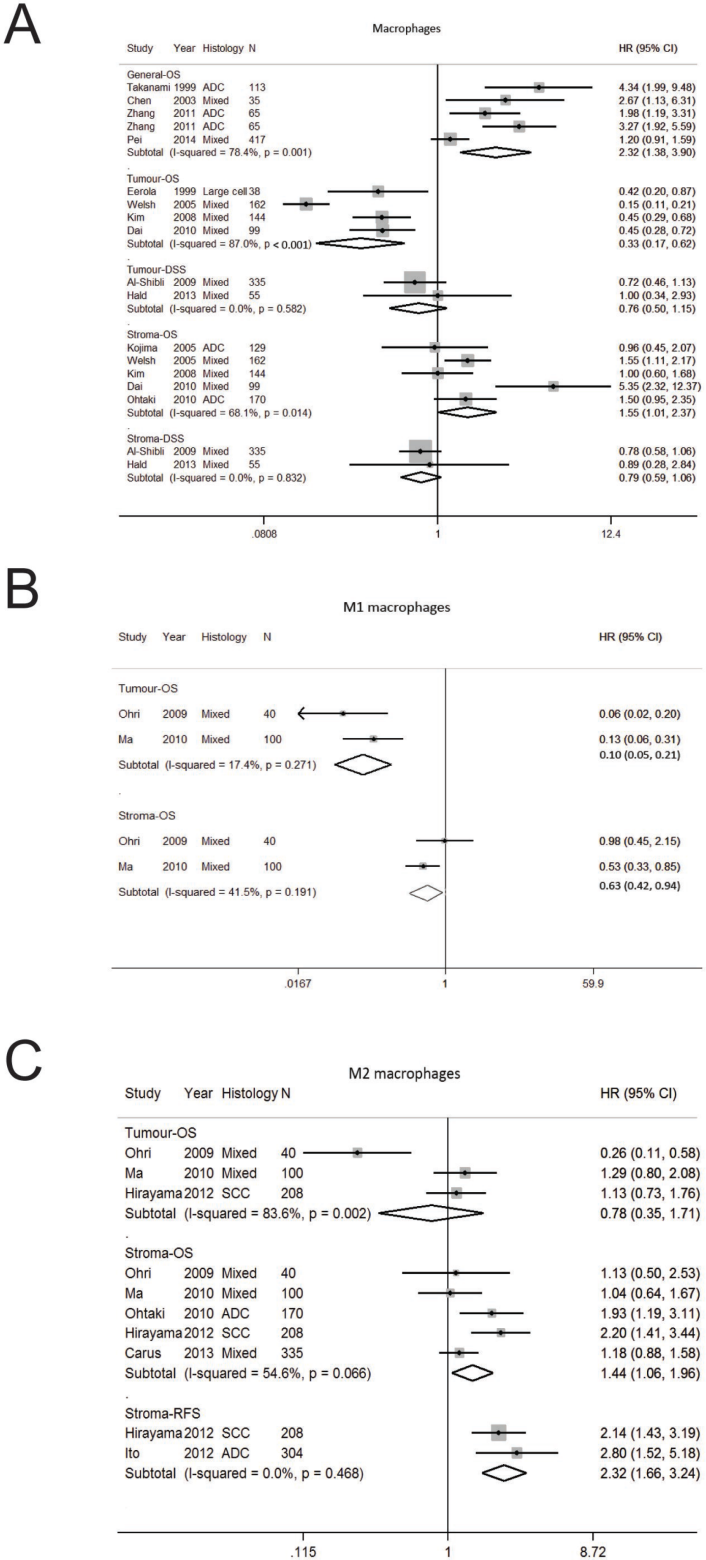
Forest plot of studies assessing (**A**) Macrophages (**B**) Macrophages M1 (**C**) Macrophages M2 and survival in patients with non-small cell lung cancer (NSCLC) stratified according to localisation (in general, tumor or stroma compartment). Adenocarcinoma, ADC; confidence interval, CI; disease specific survival, DSS; hazard ratio, HR; overall survival, OS; programmed cell death-ligand 1, PD-L1; progression free survival, PFS; relapse free survival, RFS; squamous cell carcinoma, SCC.

In our pooled analysis, increased TAMs in general had worse OS (HR 2.32; 1.38–3.90) (Table 1, Figure 2A). When analysed according to localization, increased TAMs in the tumor compartment was associated with a better OS (HR 0.33; 0.17–0.62) whereas stromal TAM was associated with poorer OS (HR 1.55; 1.01–2.37). In terms of DSS, TAMs in the tumor (HR 0.76; 0.50–1.15) and stromal (HR 0.79; 0.59–1.06) compartments was not significant (Figure 2A). A high degree of heterogeneity was seen in studies on OS according to general (I^2^ 78.4%, *p* = 0.001) and tumor (I^2^ 87.0%, *p* < 0.001) localisation. Funnel plot analysis suggest publication bias on macrophages in general compartment whereas no bias was seen for stroma macrophages (Supplementary Figure 2A and 2B).

Distinct macrophage phenotypes have been described including M1 macrophages that induce host defense, antitumor immunity and inflammatory responses and M2 macrophages reduces inflammation, suppress antitumor immunity and promote angiogenesis [37]. Given the presence of different macrophage phenotypes, we determined the prognostic effect of M1 and M2 macrophages (Table 1, Figure 2B, 2C) and found M1 macrophages was associated with improved OS in the tumor (HR 0.10; 0.05–0.19) stromal M1 and stroma (HR 0.63; 0.42–0.94). Whilst tumor M2 macrophages was not significant for OS, stroma M2 macrophages was associated with a worse OS (HR 1.44; 1.06–1.96) and RFS (HR 2.32; 1.66–3.24).

### Neutrophils

Neutrophils, a key effector immune cell, has a complex role in tumorigenesis [51]. After screening, four full text papers were reviewed [49, 52–54] but no studies were selected for pooled analysis. One study was excluded as neo-adjuvant chemotherapy was administered in 9% of patients [54] and three other studies were in a single stratum [49, 52, 53] (Supplementary Table 1, Supplementary Figure 1E). In the first study by Carus *et al*, increased neutrophils in the tumor and stroma was not associated with RFS or OS [49] whereas in the second study, increased tumor associated neutrophils (TAN) was associated with a poorer DFS [52]. In the third study, high intratumoral TANs was a positive prognosticator for DSS in SCC NSCLC whereas TAN was associated with worse DSS [53].

Tumor-associated neutrophils have a dual function characterized by the N1 and N2 phenotype in a context-dependent process. N1 neutrophils have an anti-tumor phenotype through its interaction with T cells whereas the N2 phenotype promotes tumor growth [55]. Future studies examining the prognostic role of tumor-associated neutrophils in NSCLC should take into account the distribution of N1 and N2 phenotypes within the tumor microenvironment.

### T cells, CD3 positive

**Figure 3:**
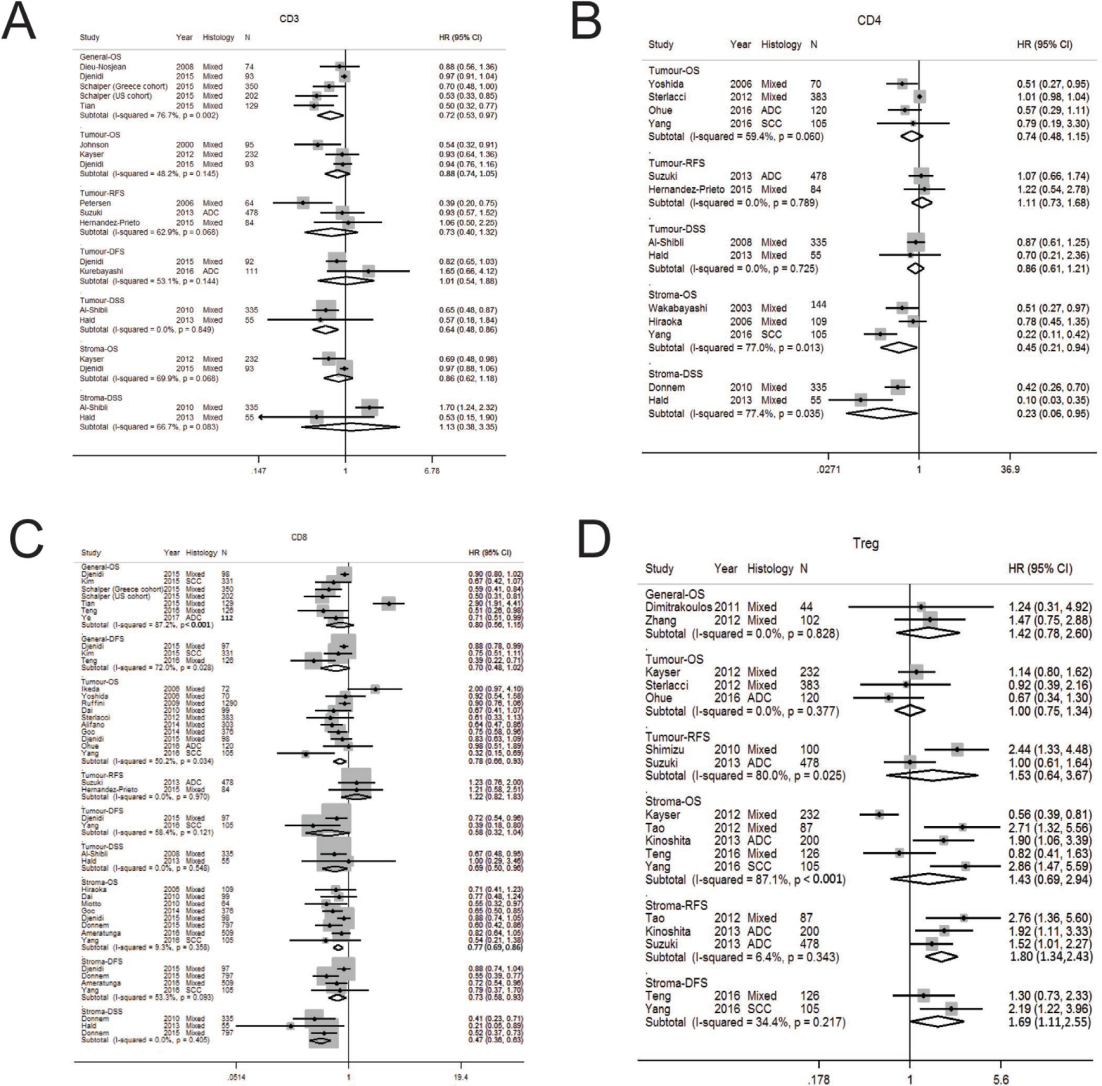
Forest plot of studies assessing (**A**) CD3+ T cells, (**B**) CD4+ T cells, (**C**) CD8+ T cells, (**D**) FOXP3+ regulatory T cells (Treg) and survival in patients with non-small cell lung cancer (NSCLC) stratified according to localisation (in general, tumor or stroma compartment). Adenocarcinoma, ADC; confidence interval, CI; Hazard ratio, disease specific survival, DSS; hazard ratio; HR; overall survival, OS; programmed cell death-ligand 1, PD-L1; progression free survival, PFS; relapse free survival, RFS; squamous cell carcinoma, SCC.

Twelve studies were analysed (Supplementary Tables 1, 2, Supplementary Figure 1F) [21, 24, 52, 56–64]. Elevated CD3+ T cells in general compartment was associated with improved OS (HR 0.72; 0.53–0.97) (Table 1, Figure 3A). When analysed according to localisation, increased tumor CD3+ T cells was associated with longer DSS (HR 0.64; 0.48–0.86) and suggestive for better OS (HR 0.88; 0.74–1.05) but not for RFS (HR 0.73; 0.40–1.32) (Table 1). Stromal CD3+ T cells was not associated with survival outcomes and may be suggestive of poorer DSS (HR 1.13) or DFS (HR 1.2), although results were not statistically significant. A high degree of heterogeneity was seen in studies on OS in the general compartment (I^2^ 70.6%, *p* = 0.002). Funnel plot suggest potential publication bias with smaller studies with favourable OS in general compartment being reported (Supplementary Figure 2C).

### T cells, CD4 positive

Eleven studies were analysed (Supplementary Tables 1, 2, Supplementary Figure 1G) [23, 24, 60, 64–71]. Study quality was generally good with an average score of 5.1 (Supplementary Table 1). CD4+ T cells in the general or tumor compartment had no influence on OS, RFS or DSS (Table 1, Figure 3B). In contrast, CD4+ T cells in the stroma compartment was associated with better OS (HR 0.45; 0.21–0.94) and DSS (HR 0.23; 0.06–0.95). Significant heterogeneity was seen in studies in the stromal compartment for OS (I^2^ 77.0%, *p* = 0.013) and DSS (I^2^ 77.4%, *p* = 0.035).

### T cells, CD8 positive

Twenty-three studies underwent pooled analysis (Supplementary Table 1, Supplementary Figure 1H) [23, 24, 29, 31, 32, 60–64, 66–78]. Although CD8+ T cells in the general compartment was not associated with OS (HR 0.80; 0.56–1.15) or DFS (HR 0.70; 0.48–1.02), when analysed according to tumor or stroma compartment, the prognostic value of CD8+ T cells could be appreciated (Table 1, Figure 3C). CD8+ T cells in the tumor compartment was associated with better prognosis in terms of OS (HR 0.78; 0.66–0.93) and DSS (HR 0.69; 0.50–0.96) but not for DFS (HR 0.58; 0.32–1.04) or RFS (HR 1.22; 0.82–1.83). Similarly, stromal CD8+ T cells conferred an improved OS (HR 0.77; 0.69–0.86), DFS (HR 0.73; 0.58–0.93) and DSS (HR 0.47; 0.36–0.63). Heterogeneity was low for most analytic strata. Funnel plot suggest generally no publication bias (Supplementary Figure 2D–2F).

### T cells, regulatory

Regulatory T cells (Tregs) are a subpopulation of CD4+ CD25+ T lymphocytes that inhibit anti-tumor immunity by promoting immune tolerance through direct suppressive functions on T cells or by secreting immunosuppressive cytokines such as IL-10 and TGF-b [79]. Tregs are purported to express and functionally depend on the transcription factor forkhead box protein P3 (FoxP3). As such many studies commonly use FoxP3 as a single marker for Tregs. Eleven studies (n=1977 patients) were reviewed (Supplementary Table 1 and 2, Supplementary Figure 1I). [23, 59, 60, 70, 71, 77, 80–84]. General Tregs infiltration was not associated with OS (HR 1.42; 0.78–2.60) (Table 1, Figure 3D) whereas tumor Tregs was not associated with OS (HR 1.00; 0.75–1.34) or RFS (HR 1.53; 0.64–3.67). Stromal Tregs was not associated with OS (HR1.43; 0.69–2.94), but however was associated with worse RFS (HR 1.80; 1.34–2.43) and DFS (HR 1.69; 1.11–2.55). Most analytic strata showed low heterogeneity. studies of tumoral Treg and RFS (I^2^ 80.0%, *p* = 0.025) and stromal OS (I^2^ 87.1%, *p* < 0.001). Possible publication bias was not observed in studies of stromal Treg at the extreme HR for OS (Supplementary Figure 2G).

### B cells

**Figure 4:**
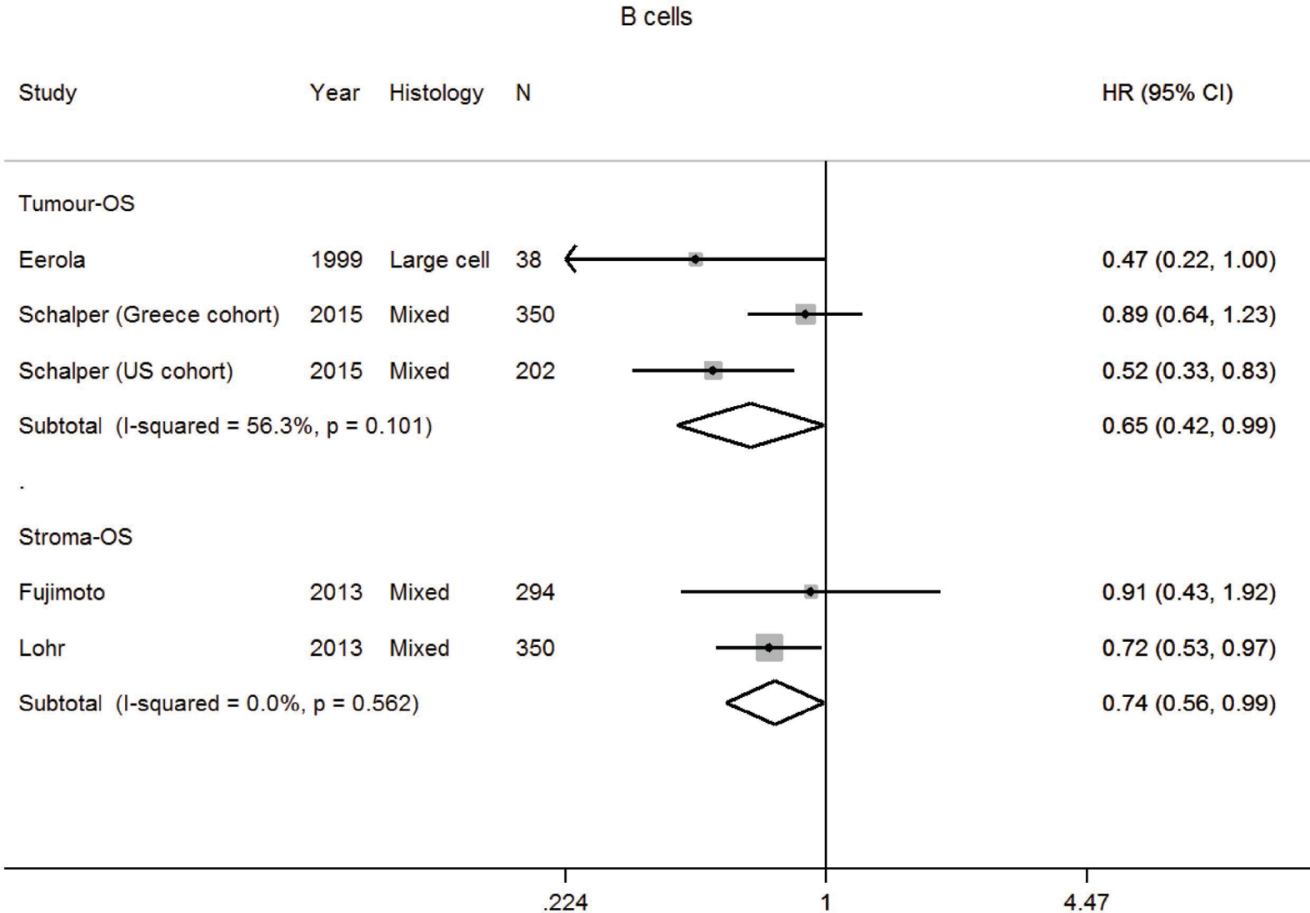
Forest plot of studies assessing B cells and overall survival (OS) in patients with non-small cell lung cancer (NSCLC) according to localisation in tumor or stroma compartment Confidence interval, CI; hazard ratio, HR.

Apart from its role in humoral immune responses, B cells have a pro- or anti-tumorigenic function [85]. After screening, four studies were analysed for OS (Supplementary Table 1 and 2, Supplementary Figure 1J) [38, 61, 86, 87]. Several studies were excluded as they had no prognostic information, insufficient information to impute HR or were the only study in an analytic stratum [21, 52, 64, 68, 88, 89]. Results of pooled analysis found B cells in the tumor and stroma was associated with an improved OS with a HR for 0.65 (0.42–0.99) and 0.74 (0.56–0.99), respectively (Table 1, Figure 4). High heterogeneity was not seen for studies of tumor and stroma B cells and OS.

### Cytotoxic T lymphocyte antigen-4

CTLA-4 is not only expressed on T cells but is also found on NSCLC tumors. Three studies were assessed in full (Supplementary Table 1, Supplementary Figure 1K). Two studies had different endpoints: OS [90] and DSS (91) therefore pooled analysis was not performed. Tumor CTLA-4 overexpression was not associated with OS [90] or DSS [91]. In the third study gene expression arrays was used and *CTLA-4* overexpression was associated worse OS [92].

### Programmed death ligand-1

**Figure 5:**
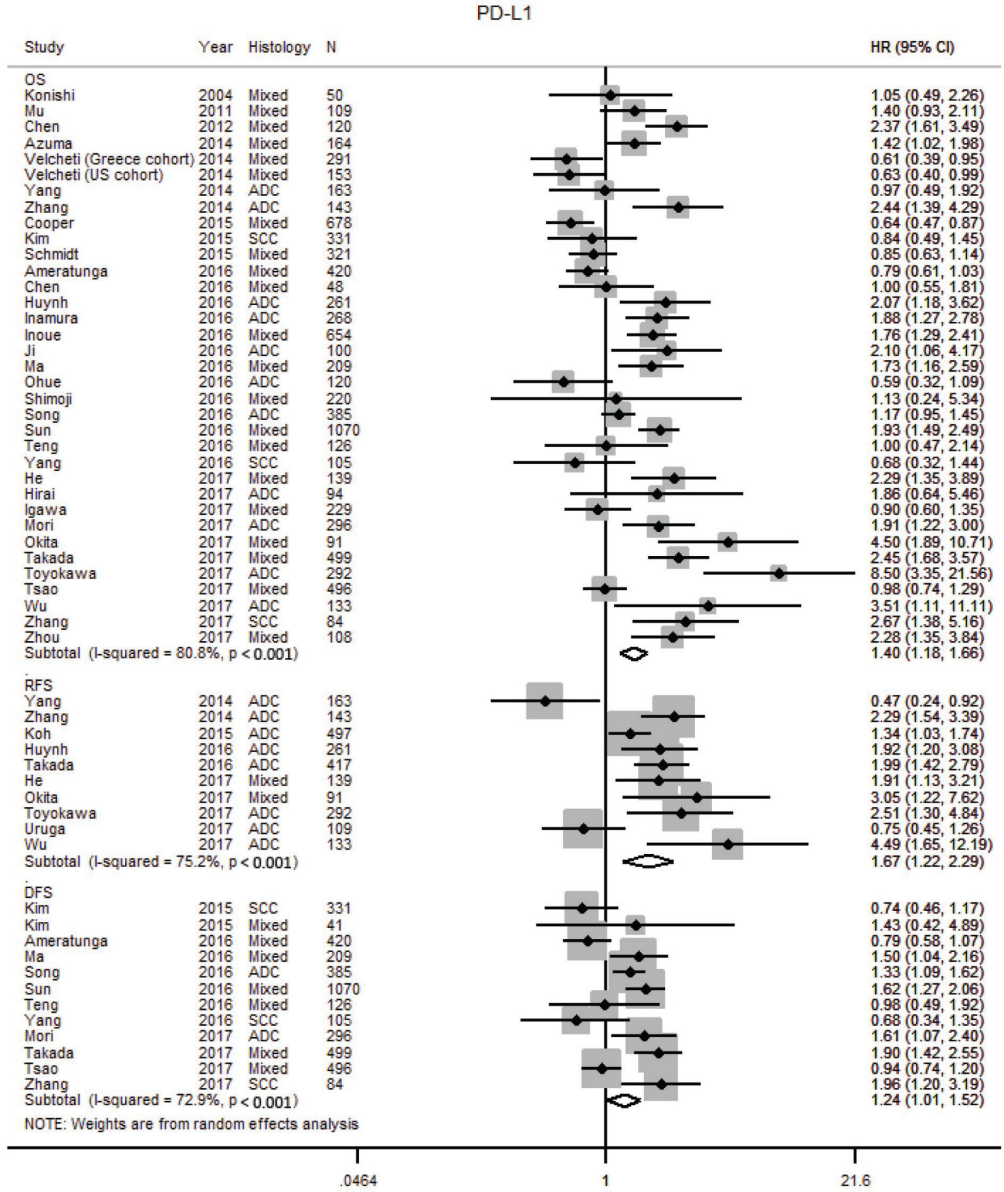
Forest plot of studies assessing tumor PD-L1 expression and survival outcomes in patients with non-small cell lung cancer (NSCLC) Adenocarcinoma, ADC; confidence interval, CI; Hazard ratio, disease free survival DFS; hazard ratio, HR; overall survival, OS; programmed cell death-ligand 1, PD-L1; progression free survival, PFS; relapse free survival, RFS; squamous cell carcinoma, SCC.

The prognostic impact of PD-L1 was reported in 38 studies with 10,034 patients (Supplementary Tables 1, 2, Supplementary Figure 1L) [30, 70, 71, 75–77, 93–124]. Two additional studies were excluded [125, 126] as there was insufficient information to calculate the HR. Our meta-analysis found tumor PD-L1 over expression was associated with worse OS (HR 1.40; 1.20–1.69), RFS (HR 1.67; 1.22–2.29) and DFS (1.24; 1.01–1.52) (Table 1, Figure 5). Heterogeneity was high in the studies for OS (I^2^ 80.8 %, *p* < 0.001) and RFS (I^2^ 75.2 %, *p* < 0.001), and moderate for DFS (I^2^ 72.9 %, *p* < 0.001). Publication bias was not observed and Egger’s test for small-study effects was not significant for studies on OS (*p* = 0.132), RFS (*p* = 0.663) and DFS (*p* = 0.656) (Supplementary Figure 2H–2J).

### Potential factors for heterogeneity

We performed sensitivity analysis and subgroup analyses for studies of each immune cell to identify potential factors responsible for the heterogeneity. Sensitivity analysis was performed by excluding studies with quality score of three or less. After excluding low quality studies, there were an inadequate number of studies of M1 macrophages for meta-analysis. Mast cells, NK cells, M2 and macrophages, CD3 lymphocytes, FOXP3+ T cells and PD-L1 meta-analysis were re-analysed and prognosis were affected for two immune cell types: OS for macrophages in general was no longer associated with poor prognosis (Supplementary Figure 3C) whereas stromal regulatory T cells was now associated with poorer prognosis (Supplementary Figure 3F). Prognostic patterns were unchanged for the other immune cells (Supplementary Figure 3). Studies of dendritic cells, CD4 and CD8 T lymphocytes were not re-analysed as all studies had quality score of at least 4.

Subgroup analyses were performed for immune cells with relatively large number of studies (≥7 studies). For this analysis, only studies of PD-L1 and CD8 satisfied this threshold. The subgroups analysed included ethnicity, publication year, sample size, sample type, and threshold for positive score. For CD8 cells, we found ethnicity, publication year, sample size and cut-off point were confounders for the association between tumour location (tumor, stroma) and OS (Supplementary Tables 4 and 5) but not for general location (Supplementary Table 3). For PD-L1 and OS, there was no confounding effect for the following variables: geographic location, publication year, sample size and histology (Supplementary Table 6). Ethnicity, histology and publication year may be a potential confounder for the association between PD-L1 and DFS (Supplementary Table 7) whereas histology may be a potential confounder for the association between PD-L1 and RFS (Supplementary Table 8). The role of histology as a confounder is limited by the small number of studies (two studies with mixed histology).

## DISCUSSION

The immune system has been implicated to have a dual role in tumorigenesis, both suppressing tumor growth through the elimination of cancer cells and also promoting tumor growth through supply of growth and survival factors. With the rapid progress being achieved in tumor immunology and the development of cancer immunotherapy approaches, an understanding of the role of immune cells in the tumor microenvironment in NSCLC may enhance these advances in immunotherapy drug development.

The role of tumor-infiltrating immune cells in NSCLC is complex and its prognostic value has been studied with variable and often conflicting results. Whilst previous meta-analyses have examined the prognostic effect of specific immune cells or immune markers such as T cells [127, 128], PD-L1 [129–135] in NSCLC, the current study to our knowledge is the first meta-analysis on the prognostic impact of NK cells, DC, MC, and macrophages. DC, NK cells, M1 macrophages, CD3+ and CD8+ T cells were found to be associated with a favourable prognosis, whereas M2 macrophages and Tregs in the stroma were associated with a worse prognosis. These results are consistent with the role of these immune cells in anti- and pro-tumor immunity, and support the pursuit of immunotherapy as a potential therapeutic modality in NSCLC [136–140].

We also confirmed localisation influenced prognosis (Table 1). DCs, NK cells, M1 macrophages and CD8+ T cells in the tumor and stroma was associated with improved prognosis. TAMs localised in the tumor had a better prognosis, whereas stromal TAMs were associated with a worse prognosis. Tumor M1 macrophages were associated with a better prognosis, whereas stromal M2 macrophages were associated with poorer OS, consistent with our understanding of the function of M1 and M2 macrophages [37]. These findings highlight the prognostic importance of both the immune cell phenotype (M1 or M2) as well as immune cell localisation in the tumor microenvironment, further emphasising the importance of a full understanding of the complexity of the cellular interactions within the tumor microenvironment.

The prognostic effect of immune cells has been reported in other tumors. Increased mast cells are associated with poorer prognosis in colorectal cancer (CRC) [141], malignant melanoma [142], pancreatic adenocarcinoma [143] and improved prognosis in malignant mesothelioma [144], ovarian cancer [145], and breast cancer [146]. Increased NK cells have been reported to be associated with an improved prognosis in gastric carcinoma [147, 148], CRC [149], and laryngeal cancer [150]. High CD3+ TILs have been associated with an improved OS in NSCLC [128], gastric [151], breast [152] and hepatocellular carcinoma (HCC) [153]. CD8+ T cells infiltration have been associated with a favourable prognosis in breast [152], ovarian [154], gastric [151], CRC [155] and HCC [153]. High FoxP3+ Tregs was associated with worse OS in cervical, renal cell carcinoma (RCC), melanoma, HCC, gastric and breast cancers and an improved OS in CRC, head and neck cancer (HNC), and oesophageal cancer whereas the DFS rate was lower in lung cancer [156]. B cell infiltration into the tumor stroma has been reported to be associated with different outcomes with an improved survival seen in breast [157], but reports for melanoma, prostate, HCC, ovarian and HNC [158] have been inconsistent. High intratumoral neutrophil density was associated with poorer OS for HCC and intrahepatic cholangiocarcinoma, HNC, NSCLC and RCC [159]. In the same meta-analysis, the HR for OS with NSCLC was borderline, with 95% CI of 1.0–1.35 and included one study where pre-operative chemotherapy was given [54].

A tremendous degree of heterogeneity was observed in terms of sample size (ranging from 38 to 1290 patients), geographical location of the patient population (East Asian/ non-East Asian); stage (I, I–III, I–IV), histology (adenocarcinoma or squamous cell only, or mixed histology), study methodology (sections or TMAs, antibodies used, scoring cutoffs), and survival endpoints (OS, DSS, DFS, RFS) (Table 1, Supplementary Tables 1, 2). The vast majority of studies examined mixed NSCLC histology with only a small number of studies focused adenocarcinoma or squamous cell histology only. The number of studies with East Asian varied depending on the immune cell studied. Immune cell studies published frequently from East Asia countries included PD-L1 (79%), TAMs (66.7%), regulatory T cells (64%), and MC (60%) whereas non-East Asian studies included CD3+ T cells (83%), NK cells (75%), B cells (75%) and CD8+ T cells (61%) (Supplementary Table 1). Such differences may account for prognostic differences observed between some studies. Subgroup analyses showed ethnicity, publication year, sample size and cut-off point were confounders for OS in CD8 (Supplementary Tables 4 and 5). The studies analysed were generally moderate in quality with an average of 3.8 for NK cell studies to 5.1 for CD4+ T cells studies (Supplementary Table 1). Therefore, higher quality studies are needed to validate the results.

The choice of antibodies used may be important as different subpopulations of immune cells exist with regard to its maturation, differentiation and state of activation. This is exemplified by the various antibody clones used to detect DCs: S100 in earlier studies [26, 27], subsequently CD1a [24, 28, 30] or CD83 [29] and more in recent studies CD208 [32, 58]. The choice of antibodies used may be important as different subpopulations of dendritic cells exist in regard to its maturation, differentiation and state of activation. Mature DC has T cell co-stimulatory molecules that induce immune reactions, whereas inactivated DCs lack such T cell stimulating ability. The use of S100 IHC Ab is controversial as its expression in DC is not specific [160]. CD83 and CD208 are markers expressed in mature DCs; whereas CD1a is expressed in immature DCs [161]. As a result, the prognostic effect of DCs in the general compartment may be affected the maturation of DC: two studies using mature DC marker were associated with favorable OS [26, 32] whereas the study of immature DC (CD1a) was associated with worse OS [30]. Similarly, in a study examining the role of DC maturation status in patients with breast cancer, CD83 expression was prognostic for overall and relapse free survival whereas CD1a, a marker of immature DC, was not [162]. Future prognostic studies should be conducted with mature DC markers localized to the tumor center.

The number of studies analyzed according to the same tissue localization of immune cell and clinical outcome was small, further limiting our ability to draw firm conclusions (Table 1). Further studies are also required to define the prognostic role of neutrophils, CTLA-4 expression in tumor cells and PD-L1 expression in immune cells.

Tumor infiltrating lymphocytes (TILs) as a whole has been reported recently in a meta-analysis to be associated with an improved PFS [127]. However, as TILs are a heterogeneous population comprising of different T cell subsets, we elected to focus on the individual subsets of TILs separately due to their different functions in tumor microenvironment [3, 4] and thus did not analyse for the prognostic effect of tumor infiltrating lymphocytes as a group.

Our findings should be interpreted within the limitations of a meta-analysis as the data was confounded by factors such as absence of individual patient data, variation in study quality, HRs calculated based on the data extracted from the survival curves, differences in tissue processing, IHC staining protocols, definition of region-of-interest, scoring methodology, differences in thresholds for positivity and prognostic end points. The use of different protocols, antibodies, and scoring systems creates complexity in the interpretation of studies and applicability in clinical practice. This is especially pertinent for the detection of PD-L1, where meta-analyses studies have shown PD-L1 expression to be associated with improved outcomes in patients with NSCLC treated with immune checkpoint inhibitors [163–165]. Given the role of PD-L1 as a prognostic biomarker and, more importantly, as a predictive marker for treatment selection, further efforts are clearly required to standardise the detection of PD-L1 expression and also to determine factors of variability between IHC assays [166, 167]. Apart from to PD-L1, international efforts are also underway to standardise the assessment of tumor-infiltrating lymphocytes in NSCLC as well as other solid tumors [168, 169].

Future studies should examine the role of immune cells as a new prognostic factor in staging. Similar to developments were made in CRC [170, 171], while approaches to integrate tumor-infiltrating lymphocytes into NSCLC staging are being pursued [172]. In addition to incorporating tumor-infiltrating lymphocytes, further prospective studies using multi-immune cell panels/ multi-parametric IHC are also desirable to determine the most promising combination of immune cells as a prognostic marker in NSCLC. Recent studies of molecular tumor profiling with immune cell phenotyping [173, 174] has improved our understanding of the complex relationship between tumor and the tumor microenvironment and may lead to improvements in therapeutic outcomes in NSCLC.

## CONCLUSIONS

Our findings suggest DC, NK cells, M1 macrophages, CD8+ T cells, and B cells in the tumor and stroma are associated with an improved prognosis and stromal M2 macrophages, regulatory T cells and PD-L1 overexpression are associated with poorer prognosis in NSCLC. Future research should focus on the standardisation of immune cell detection, use of multi-immune cell panels as a prognostic biomarker, and incorporating immune cells into prognostic models.

## SUPPLEMENTARY MATERIALS FIGURES AND TABLES






