# Supplementary material for: Prognostic significance of immune cells in non-small cell lung cancer: meta-analysis
Source: Oncotarget. 2018 May 15;9(37):24801–20. doi: 10.18632/oncotarget.24835 (PMC5973851; doi:10.18632/oncotarget.24835)
Supplement: Supplementary file 1 [file oncotarget-09-24801-s001.pdf]

Prognostic significance of immune cells in non-small cell lung cancer: meta-analysis

SUPPLEMENTARY MATERIALS

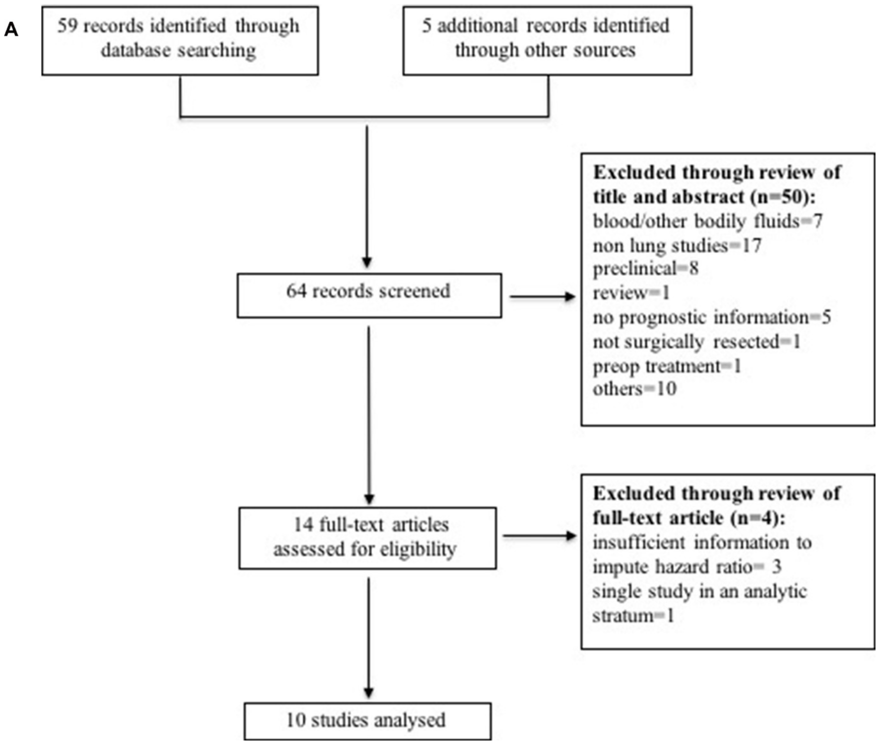

B

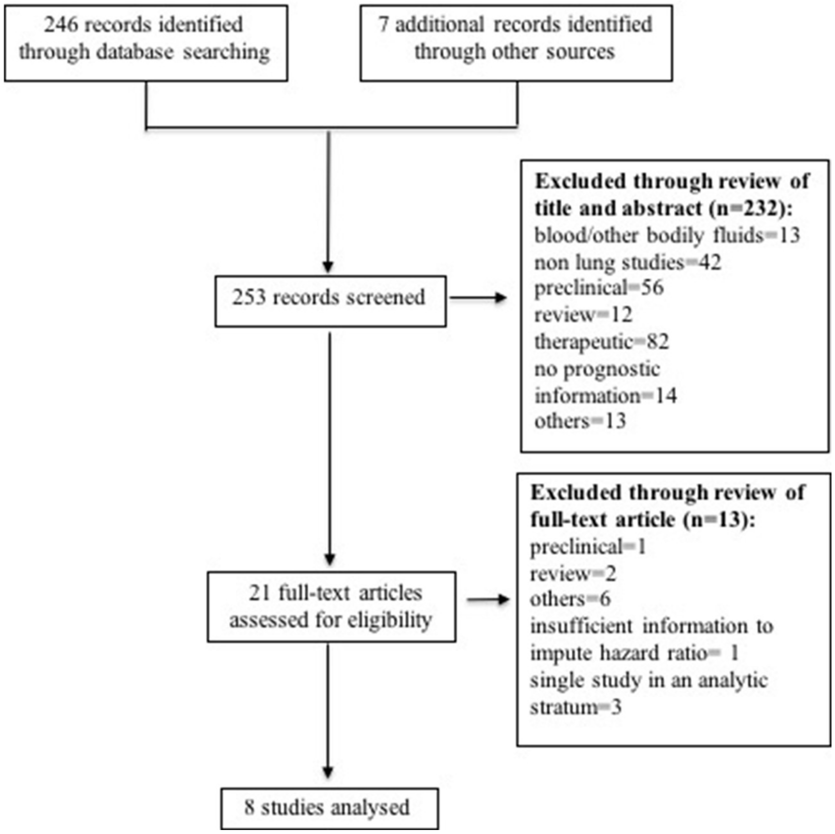

246 records identified through database searching
7 additional records identified through other sources
253 records screened
Excluded through review of title and abstract (n=232):
blood/other bodily fluids=13
non lung studies=42
preclinical=56
review=12
therapeutic=82
no prognostic information=14
others=13
21 full-text articles assessed for eligibility
Excluded through review of full-text article (n=13):
preclinical=1
review=2
others=6
insufficient information to impute hazard ratio= 1
single study in an analytic stratum=3
8 studies analysed

C

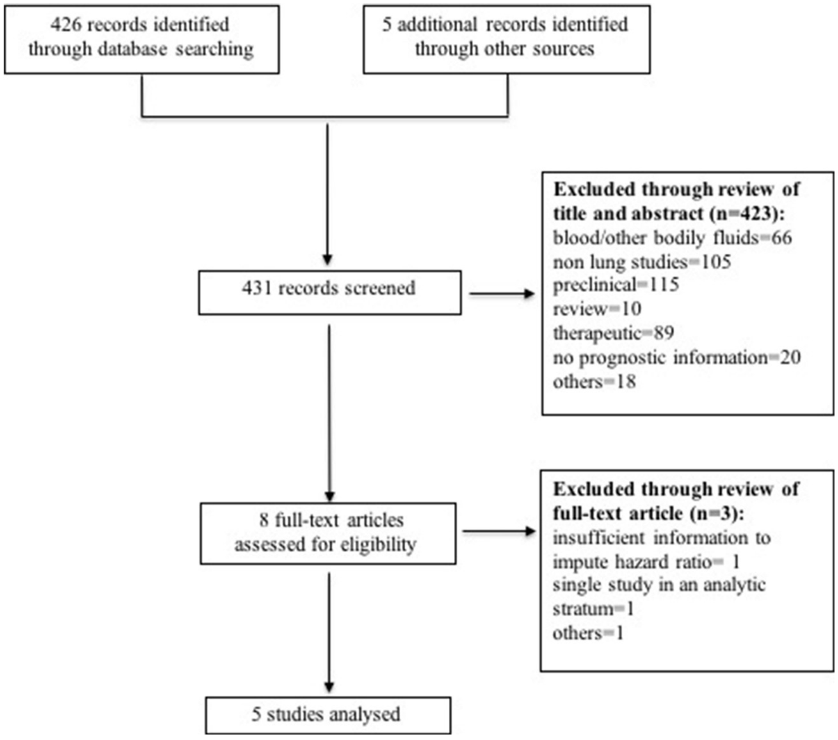

426 records identified through database searching
5 additional records identified through other sources
431 records screened
Excluded through review of title and abstract (n=423):
blood/other bodily fluids=66
non lung studies=105
preclinical=115
review=10
therapeutic=89
no prognostic information=20
others=18
8 full-text articles assessed for eligibility
Excluded through review of full-text article (n=3):
insufficient information to impute hazard ratio= 1
single study in an analytic stratum=1
others=1
5 studies analysed

D

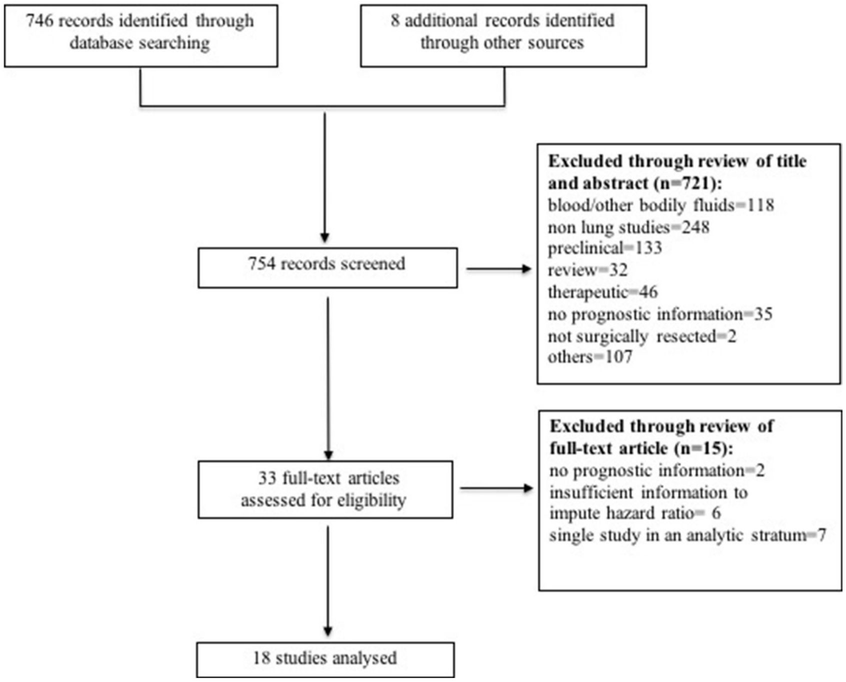
746 records identified through database searching
8 additional records identified through other sources
754 records screened
Excluded through review of title and abstract (n=721):
blood/other bodily fluids=118
non lung studies=248
preclinical=133
review=32
therapeutic=46
no prognostic information=35
not surgically resected=2
others=107
33 full-text articles assessed for eligibility
Excluded through review of full-text article (n=15):
no prognostic information=2
insufficient information to impute hazard ratio= 6
single study in an analytic stratum=7
18 studies analysed

E

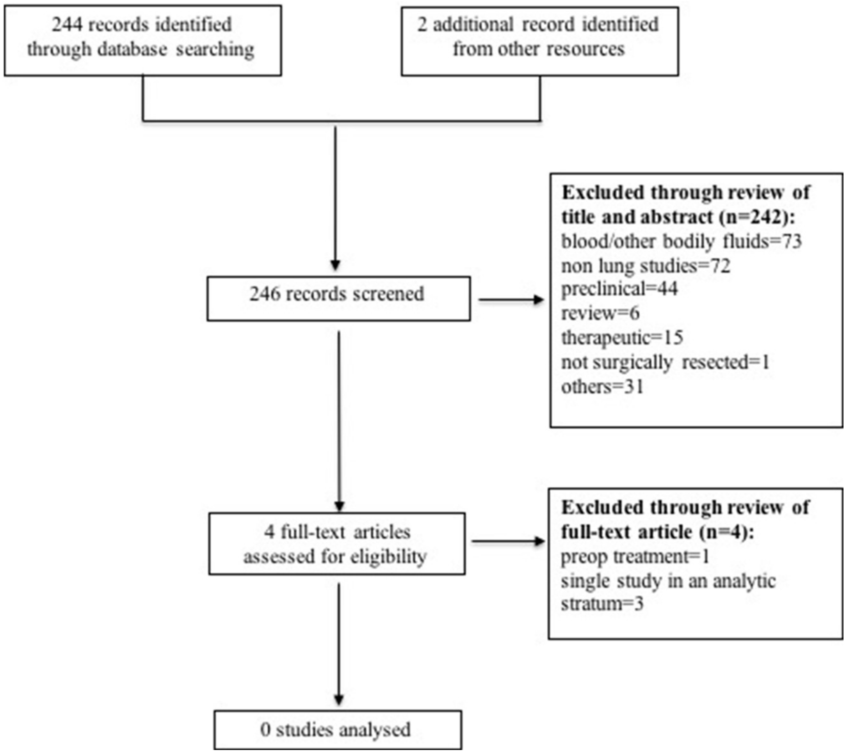
244 records identified through database searching
2 additional record identified from other resources
246 records screened
Excluded through review of title and abstract (n=242):
blood/other bodily fluids=73
non lung studies=72
preclinical=44
review=6
therapeutic=15
not surgically resected=1
others=31
4 full-text articles assessed for eligibility
Excluded through review of full-text article (n=4):
preop treatment=1
single study in an analytic stratum=3
0 studies analysed

F

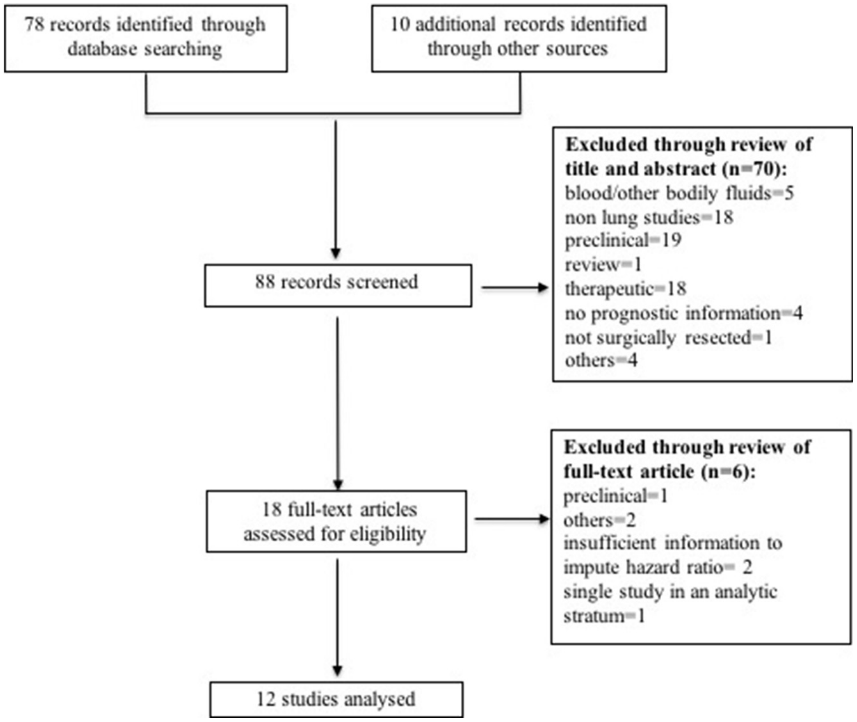
78 records identified through database searching
10 additional records identified through other sources
88 records screened
Excluded through review of title and abstract (n=70):
blood/other bodily fluids=5
non lung studies=18
preclinical=19
review=1
therapeutic=18
no prognostic information=4
not surgically resected=1
others=4
18 full-text articles assessed for eligibility
Excluded through review of full-text article (n=6):
preclinical=1
others=2
insufficient information to impute hazard ratio= 2
single study in an analytic stratum=1
12 studies analysed

G

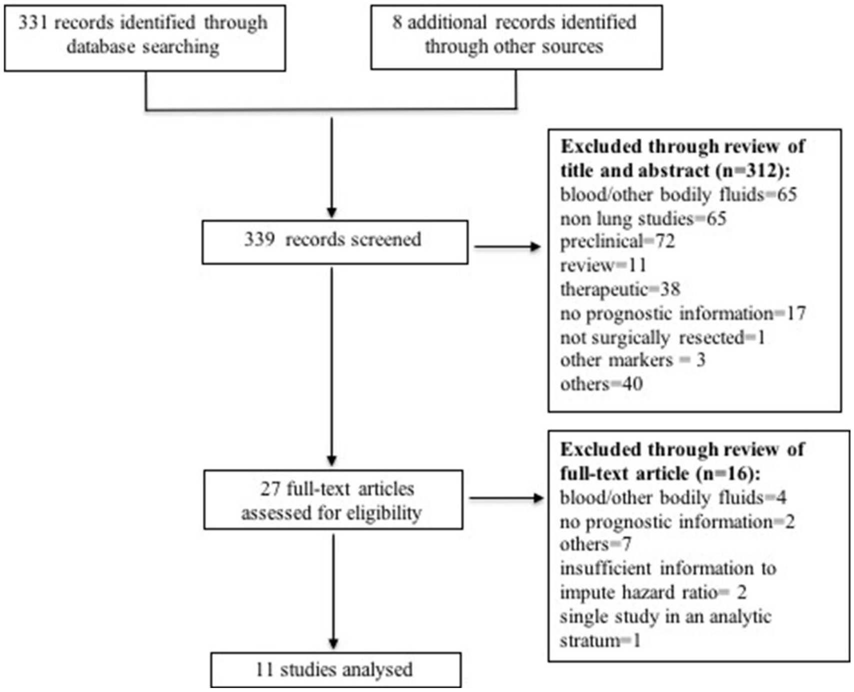
331 records identified through database searching
8 additional records identified through other sources
339 records screened
Excluded through review of title and abstract (n=312):
blood/other bodily fluids=65
non lung studies=65
preclinical=72
review=11
therapeutic=38
no prognostic information=17
not surgically resected=1
other markers = 3
others=40
27 full-text articles assessed for eligibility
Excluded through review of full-text article (n=16):
blood/other bodily fluids=4
no prognostic information=2
others=7
insufficient information to impute hazard ratio= 2
single study in an analytic stratum=1
11 studies analysed

H

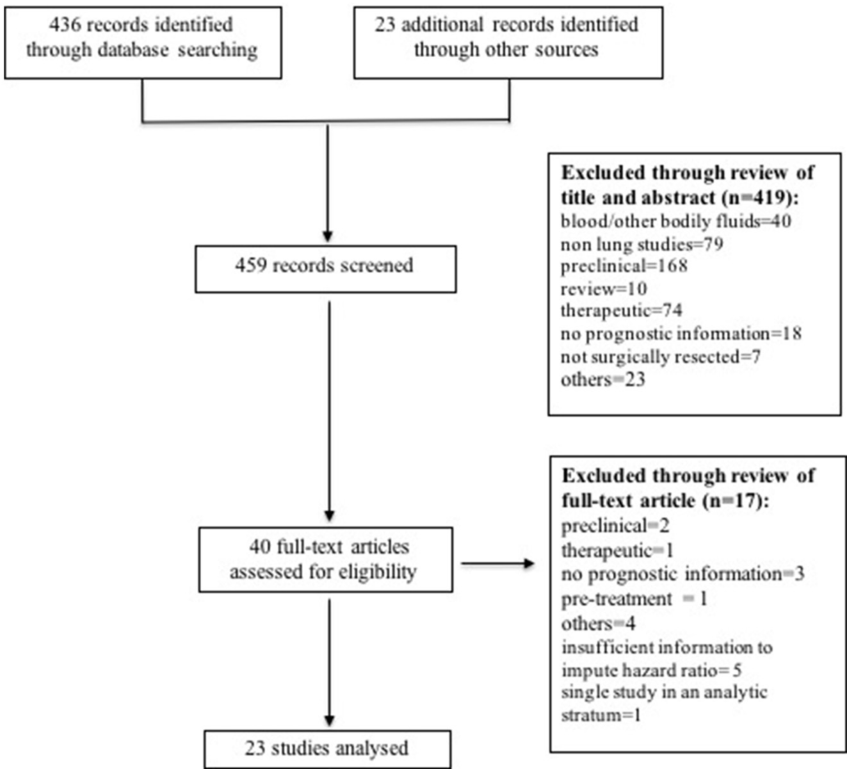
436 records identified through database searching
23 additional records identified through other sources
459 records screened
Excluded through review of title and abstract (n=419):
blood/other bodily fluids=40
non lung studies=79
preclinical=168
review=10
therapeutic=74
no prognostic information=18
not surgically resected=7
others=23
40 full-text articles assessed for eligibility
Excluded through review of full-text article (n=17):
preclinical=2
therapeutic=1
no prognostic information=3
pre-treatment = 1
others=4
insufficient information to impute hazard ratio= 5
single study in an analytic stratum=1
23 studies analysed

I

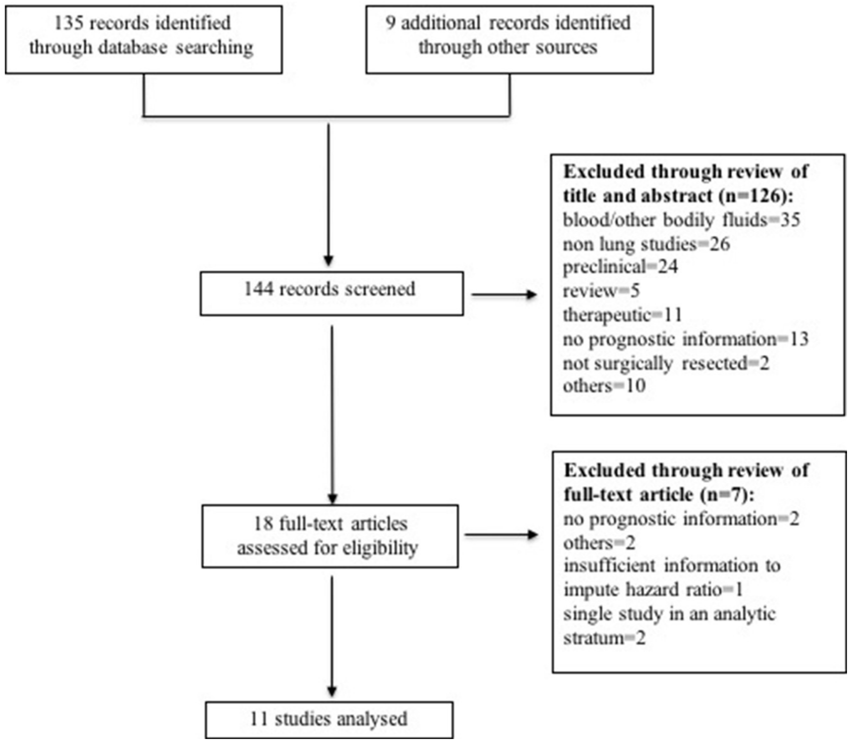
135 records identified through database searching
9 additional records identified through other sources
144 records screened
Excluded through review of title and abstract (n=126):
blood/other bodily fluids=35
non lung studies=26
preclinical=24
review=5
therapeutic=11
no prognostic information=13
not surgically resected=2
others=10
18 full-text articles assessed for eligibility
Excluded through review of full-text article (n=7):
no prognostic information=2
others=2
insufficient information to impute hazard ratio=1
single study in an analytic stratum=2
11 studies analysed

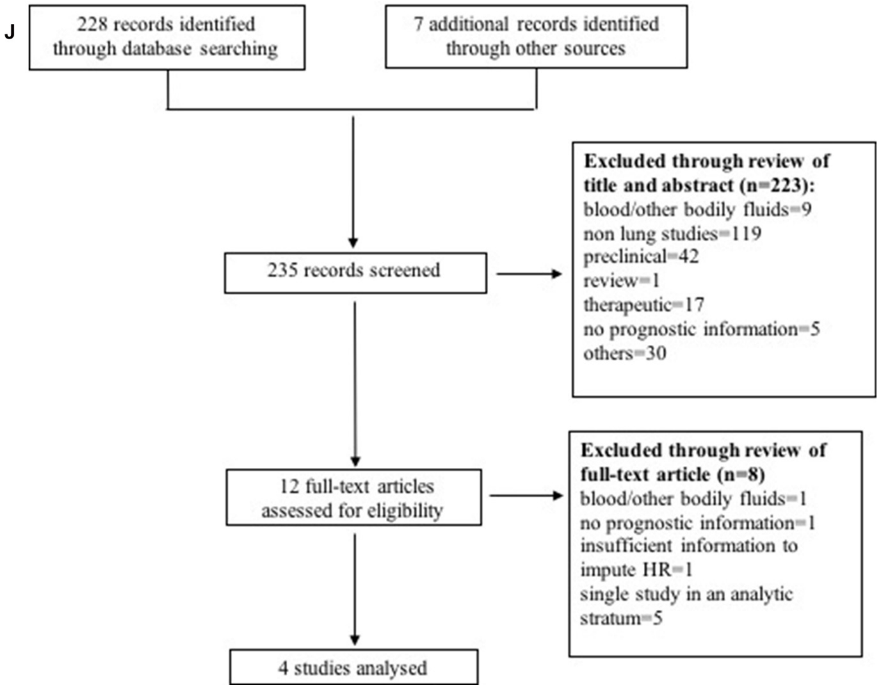
J
228 records identified through database searching
7 additional records identified through other sources
235 records screened
Excluded through review of title and abstract (n=223):
blood/other bodily fluids=9
non lung studies=119
preclinical=42
review=1
therapeutic=17
no prognostic information=5
others=30
12 full-text articles assessed for eligibility
Excluded through review of full-text article (n=8)
blood/other bodily fluids=1
no prognostic information=1
insufficient information to impute HR=1
single study in an analytic stratum=5
4 studies analysed

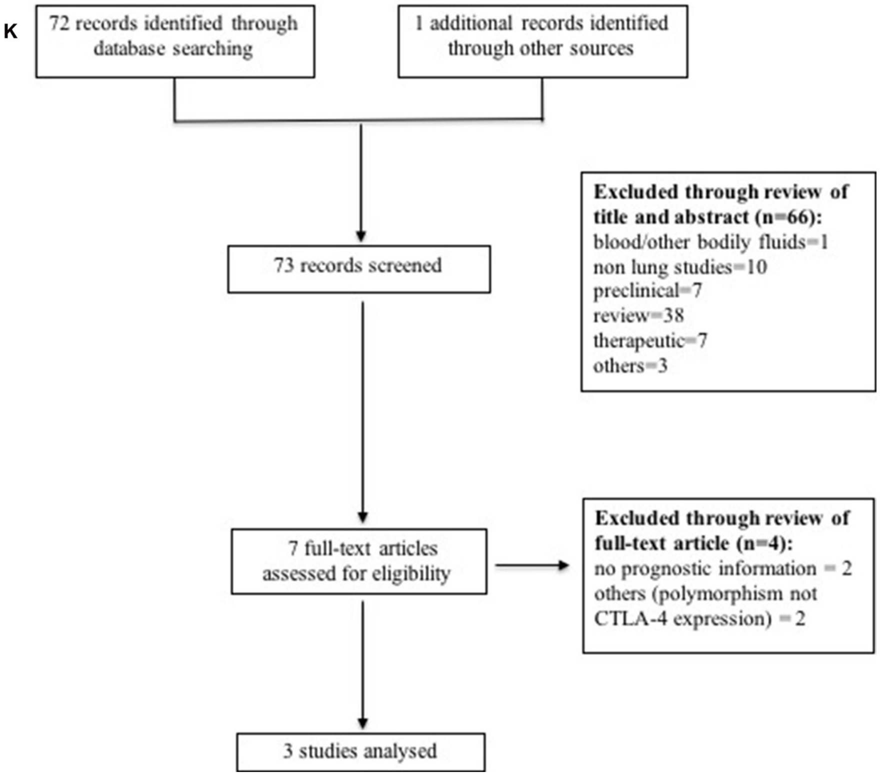
K
72 records identified through database searching
1 additional records identified through other sources
73 records screened
Excluded through review of title and abstract (n=66):
blood/other bodily fluids=1
non lung studies=10
preclinical=7
review=38
therapeutic=7
others=3
7 full-text articles assessed for eligibility
Excluded through review of full-text article (n=4):
no prognostic information = 2
others (polymorphism not CTLA-4 expression) = 2
3 studies analysed

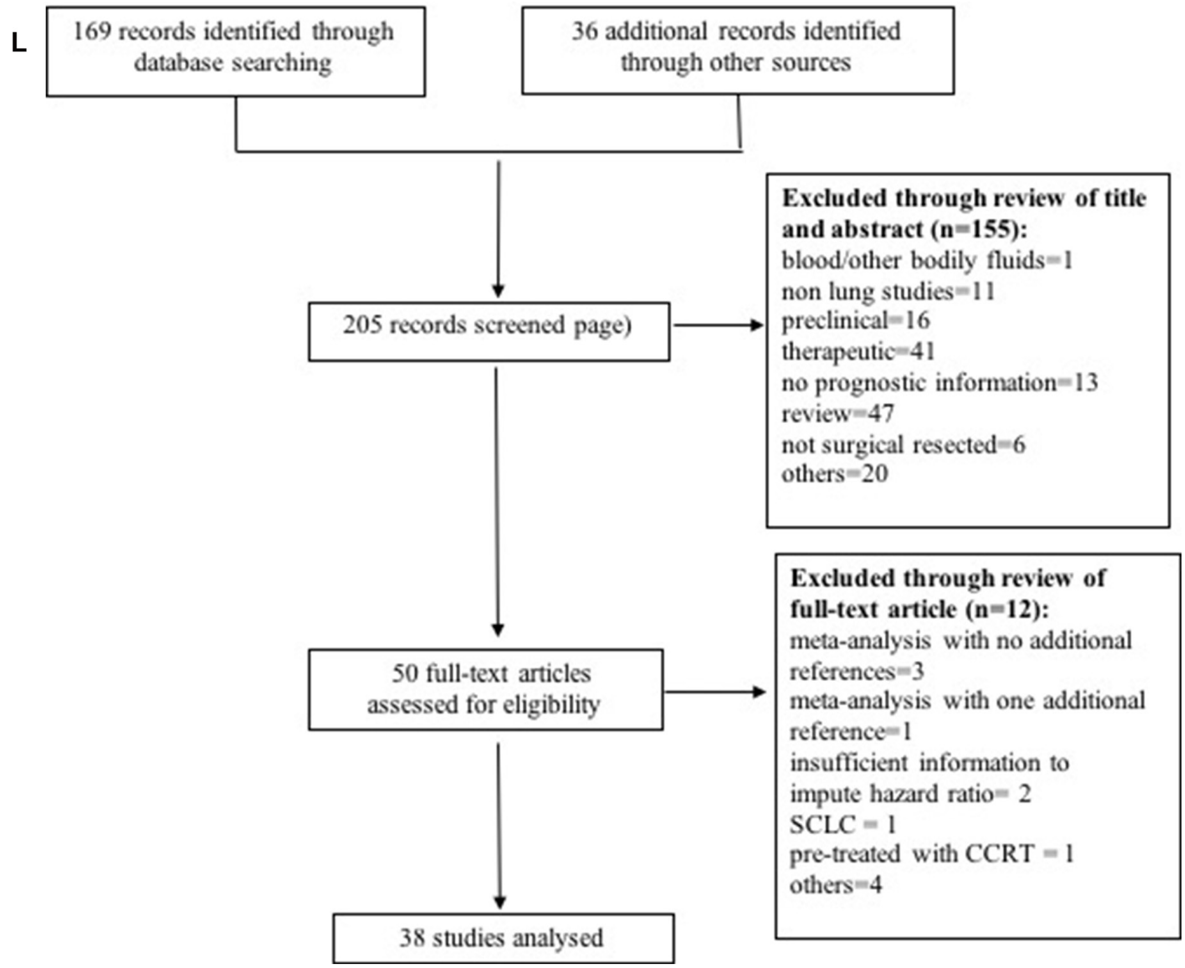

Supplementary Figure 1: Flow chart on selection of studies on immune cell and prognosis in non-small cell lung cancer (**A**) mast cells, (**B**) Dendritic cells, (**C**) Natural killer cells, (**D**) Tumor associated macrophages, (**E**) Neutrophils, (**F**) T cells, CD3, (**G**) T cells, CD4, (**H**) T cells, CD8, (**I**) T cells, Regulatory, (**J**) B cells, (**K**) Tumor CTLA, (**L**) Tumor PD-L1

A

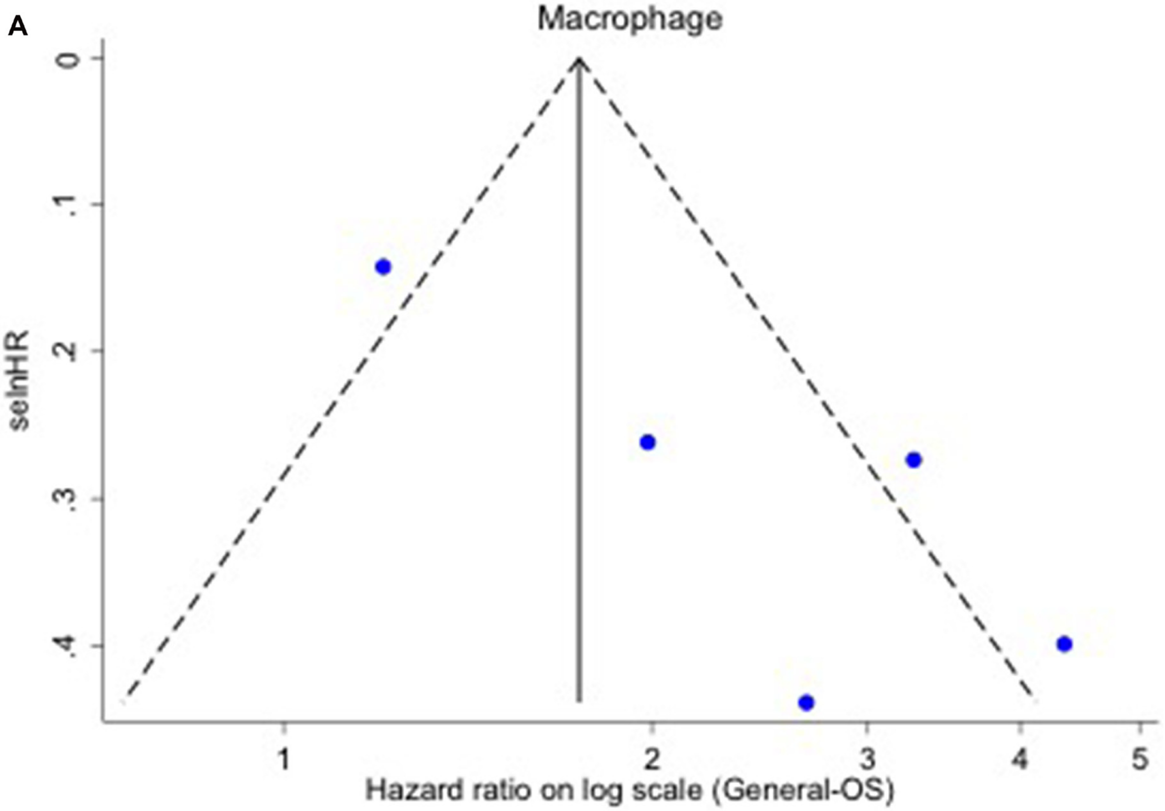
Macrophage
0
.1
.2
.3
.4
selnHR
1
2
3
4
5
Hazard ratio on log scale (General-OS)

B

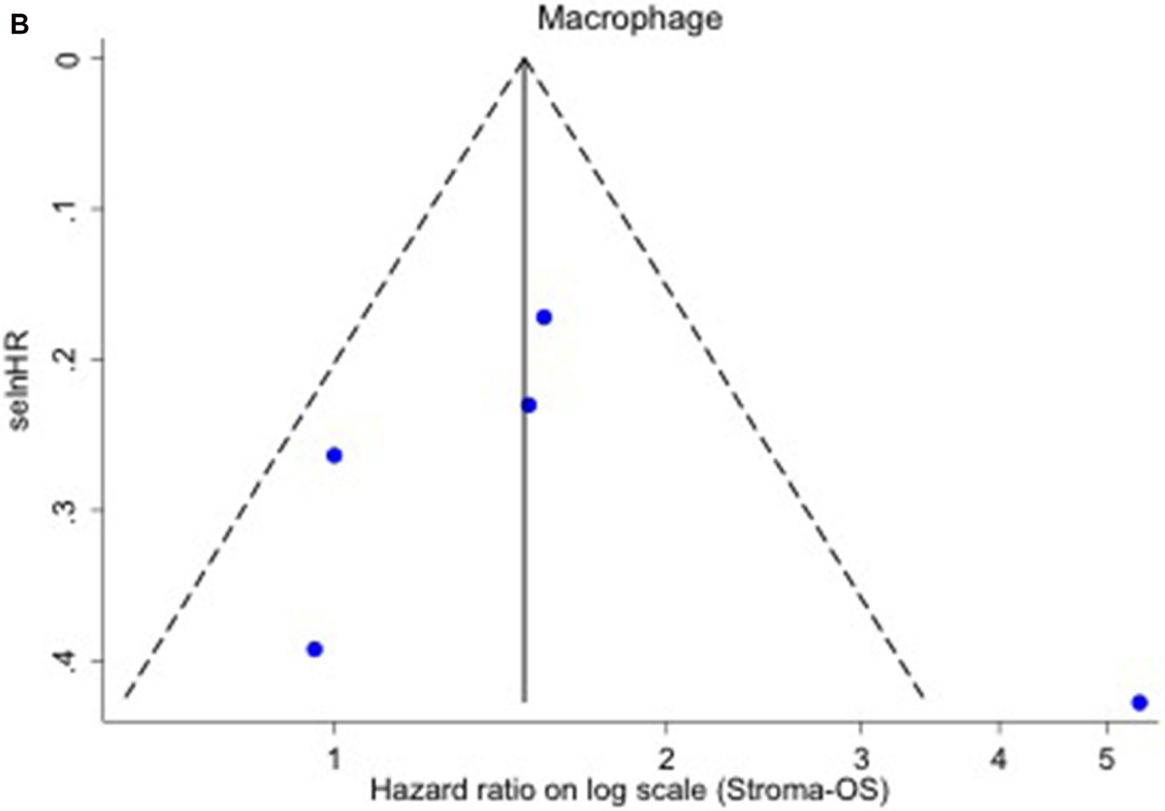
Macrophage
0
.1
.2
.3
.4
selnHR
1
2
3
4
5
Hazard ratio on log scale (Stroma-OS)

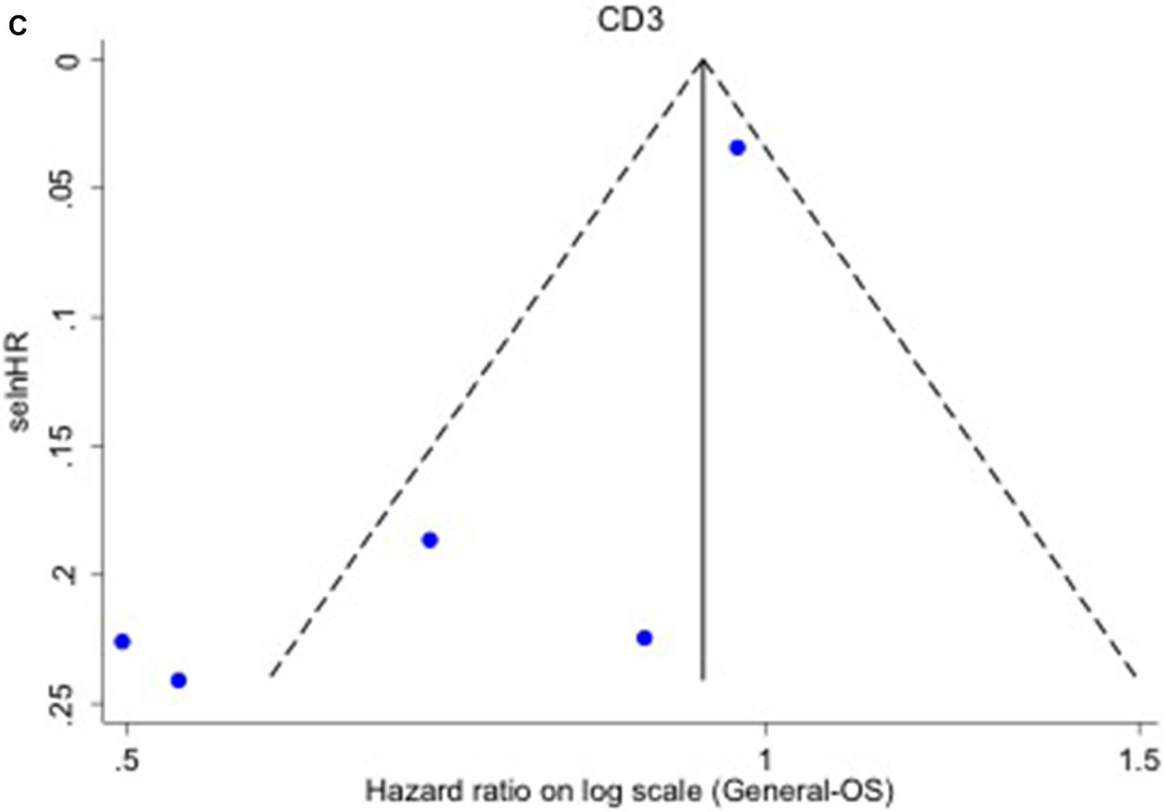
C
CD3
selnHR
0
.05
.1
.15
.2
.25
.5
1
1.5
Hazard ratio on log scale (General-OS)

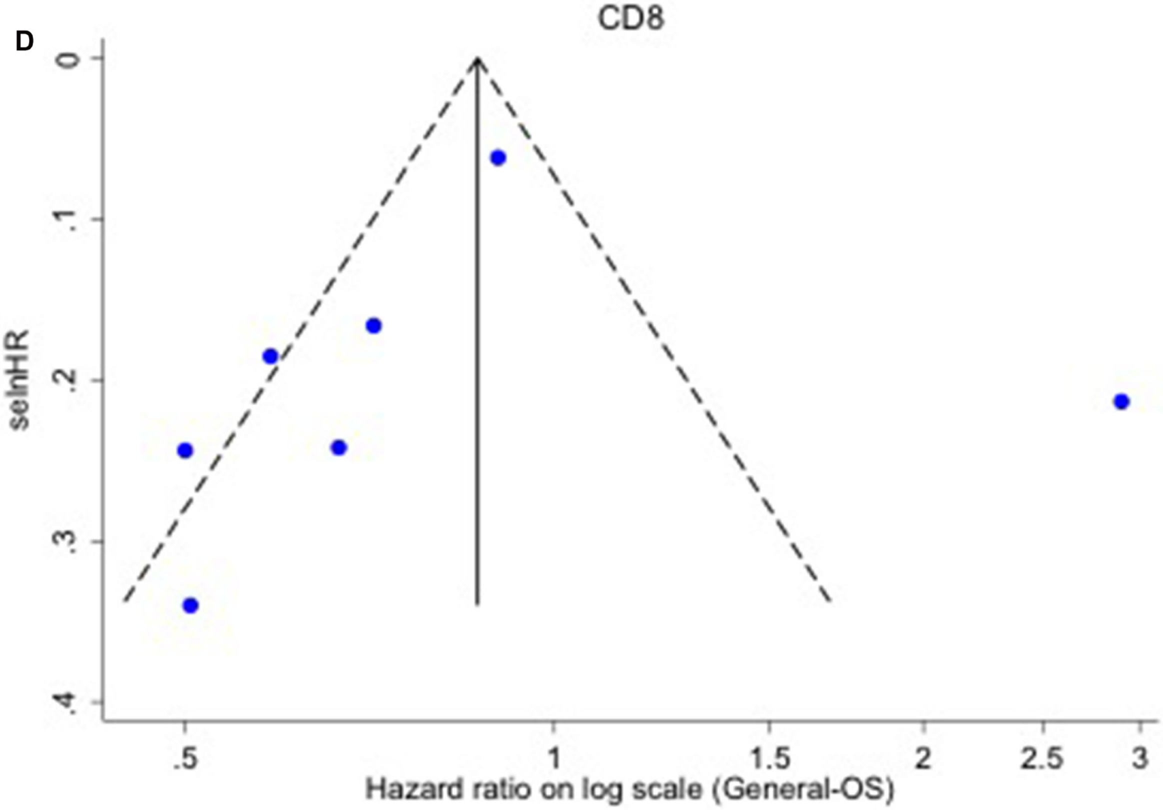
D
CD8
selnHR
0
.1
.2
.3
.4
.5
1
1.5
2
2.5
3
Hazard ratio on log scale (General-OS)

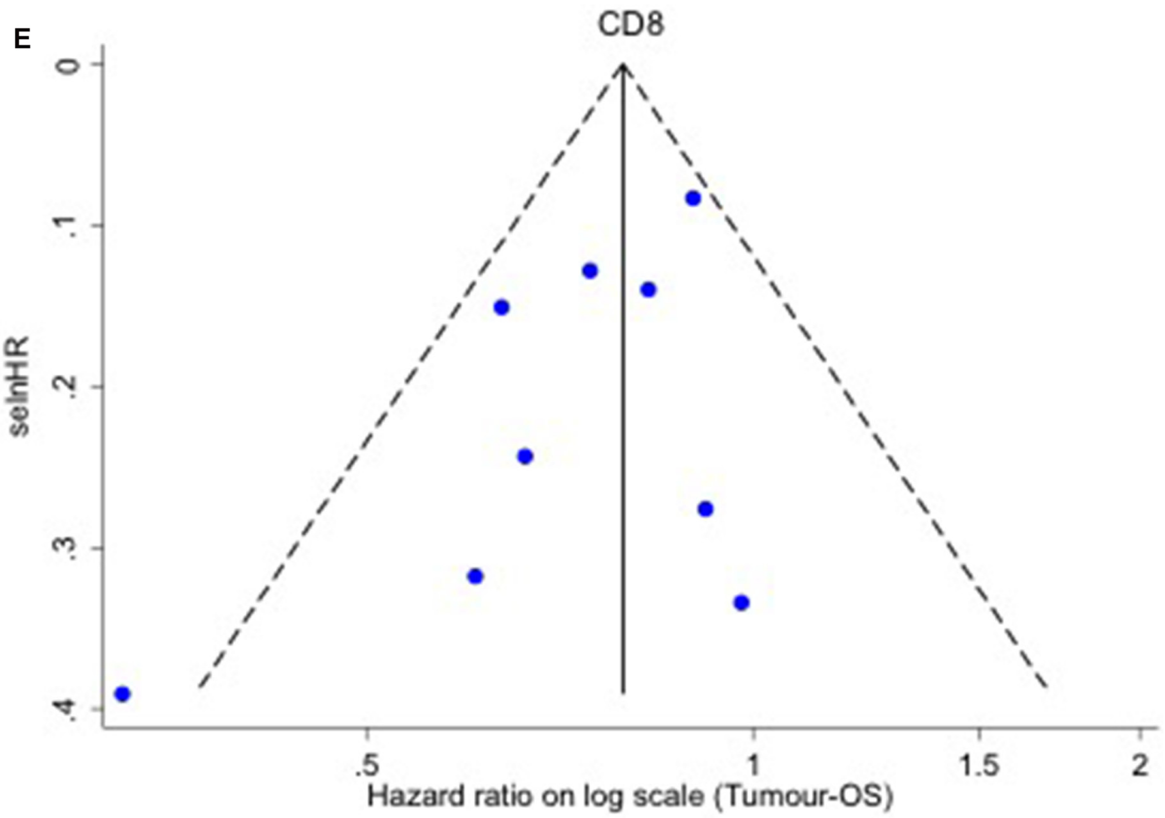

E
CD8
0
.1
.2
.3
.4
seInHR
.5
1
1.5
2
Hazard ratio on log scale (Tumour-OS)

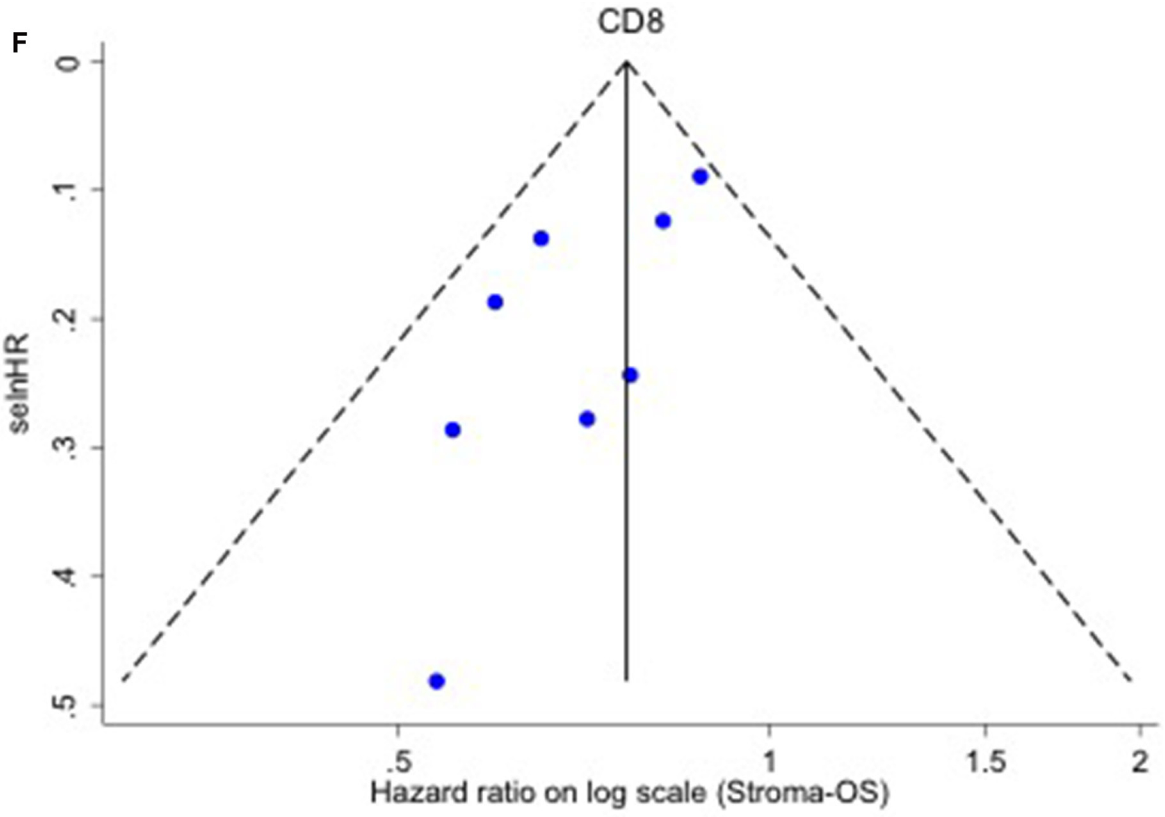

F
CD8
0
.1
.2
.3
.4
.5
seInHR
.5
1
1.5
2
Hazard ratio on log scale (Stroma-OS)

G

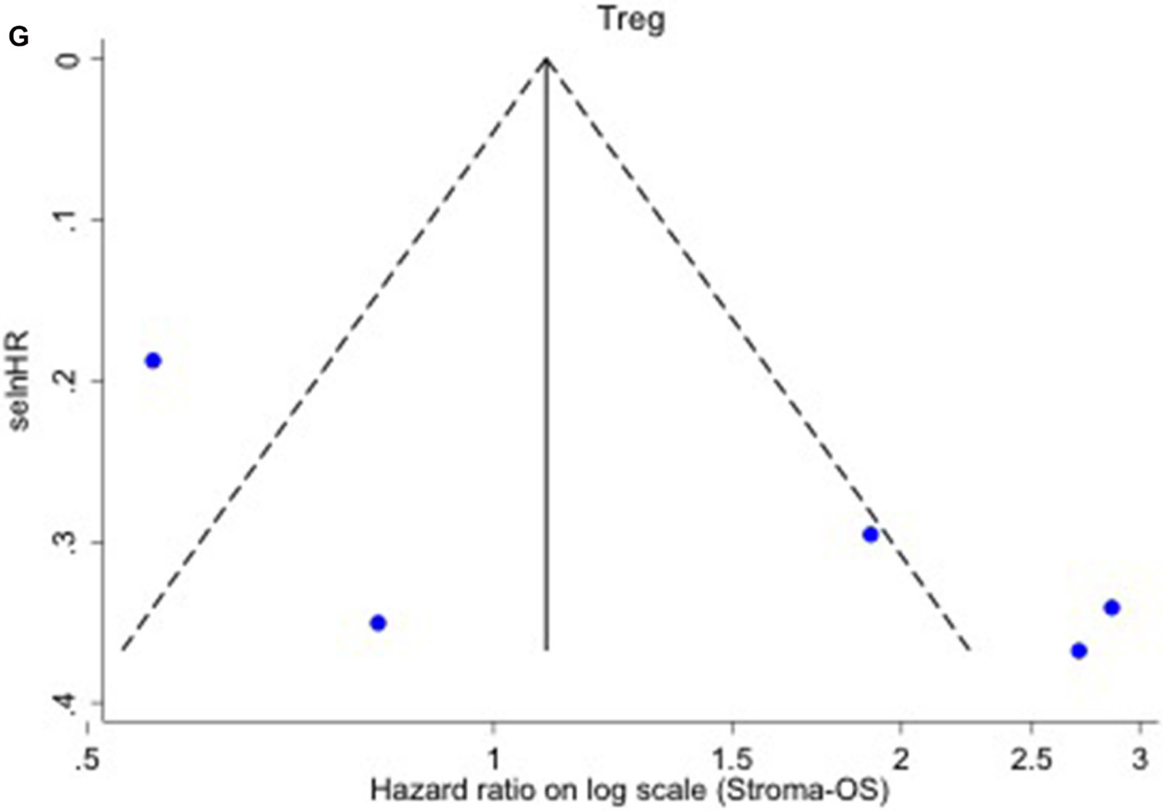
Treg
selnHR
0
.1
.2
.3
.4
.5
1
1.5
2
2.5
3
Hazard ratio on log scale (Stroma-OS)

H

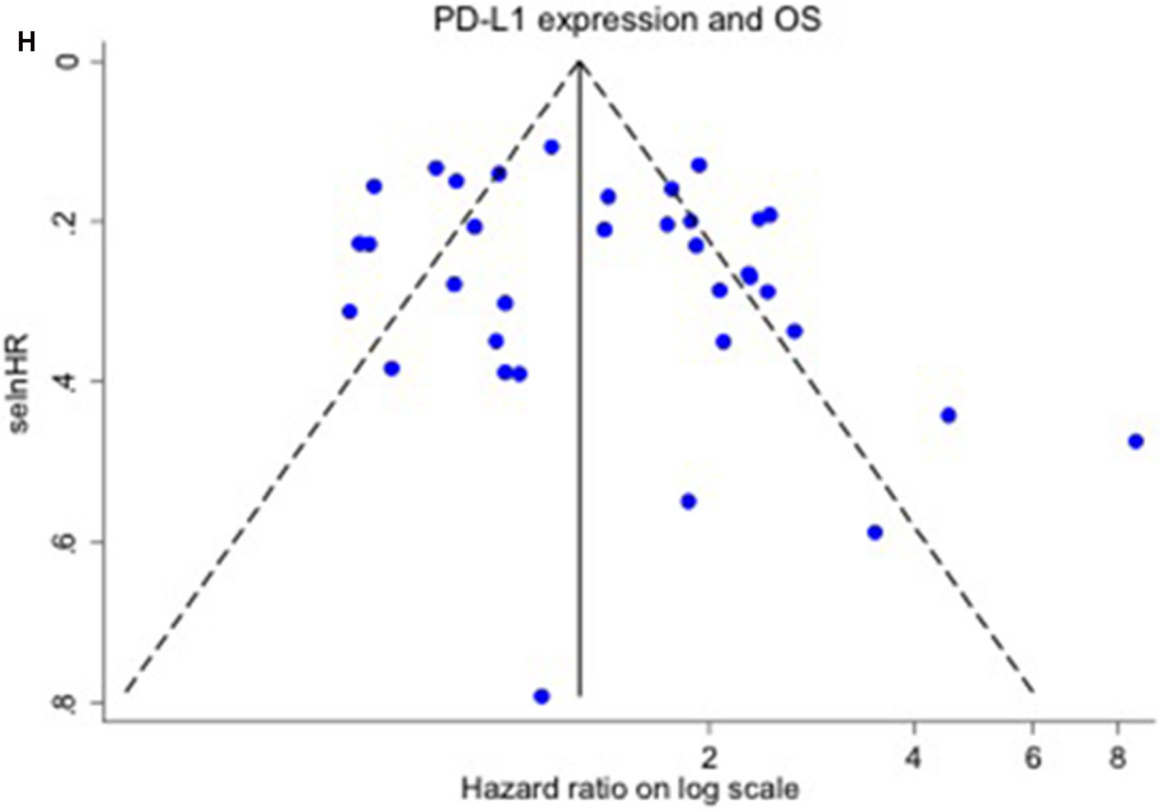
PD-L1 expression and OS
selnHR
0
.2
.4
.6
.8
2
4
6
8
Hazard ratio on log scale

I

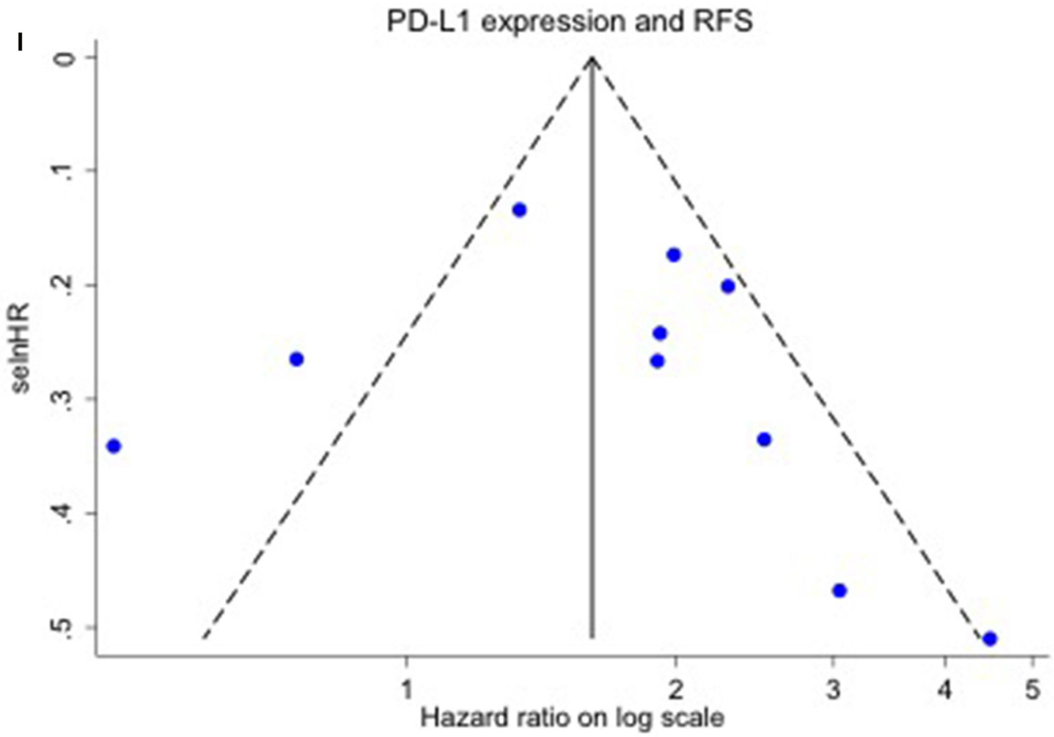

J

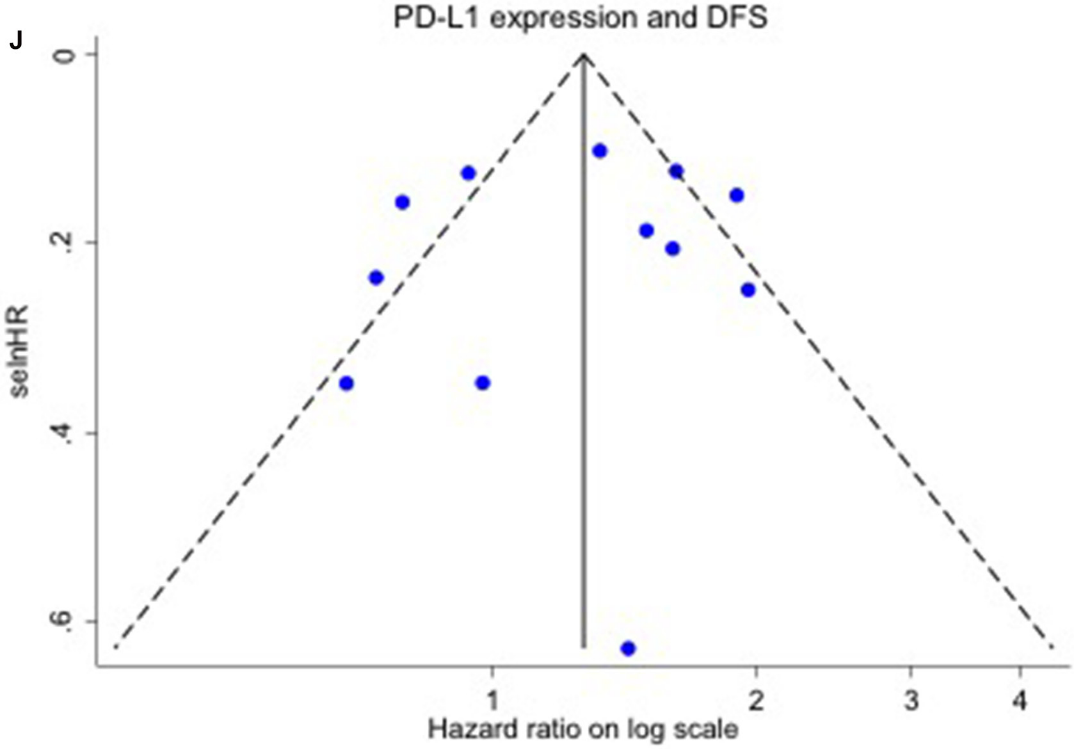

Supplementary Figure 2: Funnel plot for potential publication bias of Hazard ratio for overall survival (OS) in eligible studies (**A**) Macrophage in general (**B**) Macrophage in stroma (**C**) CD3+ T cells in general (**D**) CD8 + T cells in general (**E**) CD8+ T cells in tumor (**F**) CD8+ T cells in stroma (**G**) FOXP3+ T cells in stroma (**H**) PDL1 for OS (**I**) PDL1 for relapse free survival (RFS), PDL1 for disease free survival (DFS)

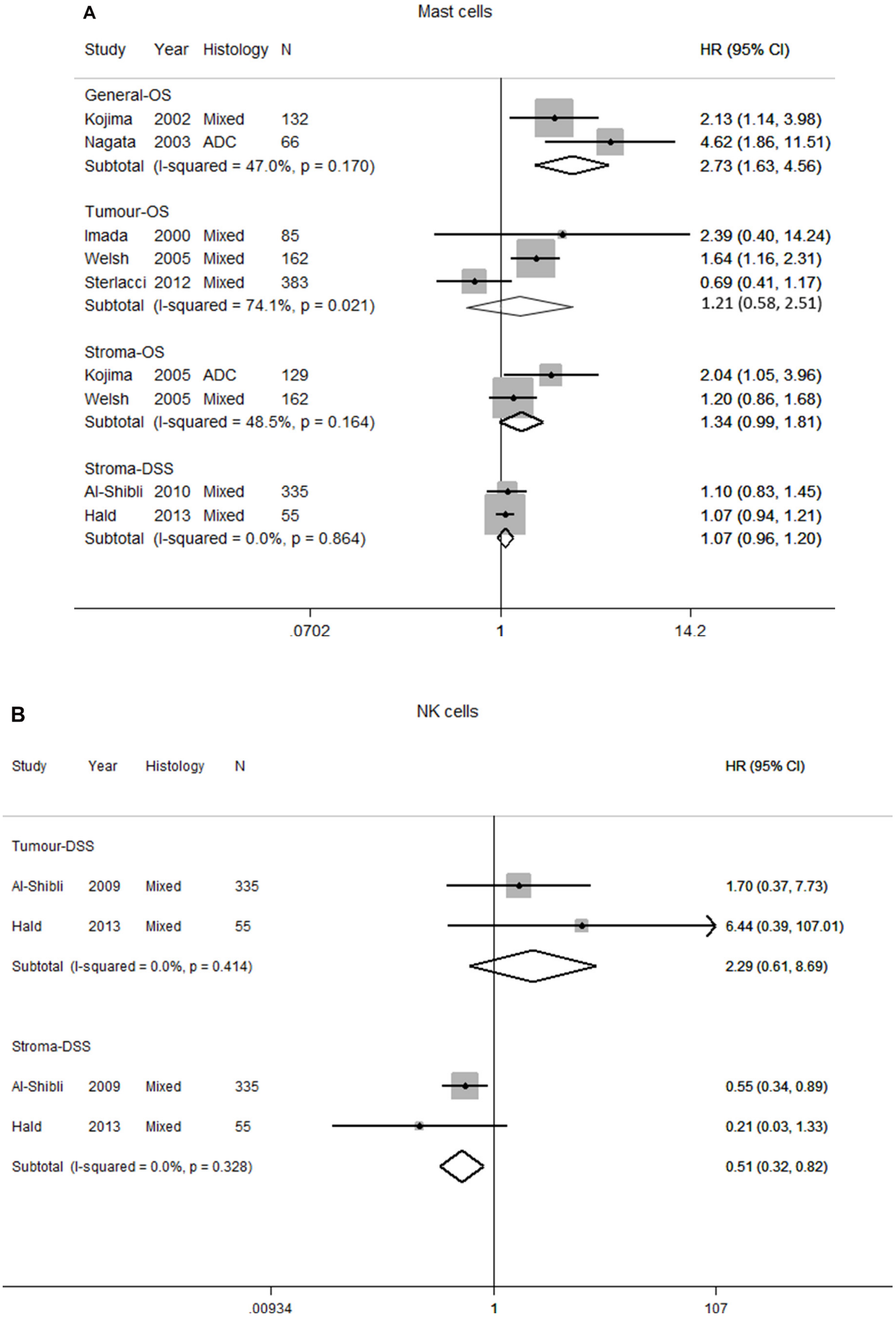
A
Mast cells
Study Year Histology N
HR (95% CI)
General-OS
Kojima 2002 Mixed 132 2.13 (1.14, 3.98)
Nagata 2003 ADC 66 4.62 (1.86, 11.51)
Subtotal (I-squared = 47.0%, p = 0.170) 2.73 (1.63, 4.56)
Tumour-OS
Imada 2000 Mixed 85 2.39 (0.40, 14.24)
Welsh 2005 Mixed 162 1.64 (1.16, 2.31)
Sterlacci 2012 Mixed 383 0.69 (0.41, 1.17)
Subtotal (I-squared = 74.1%, p = 0.021) 1.21 (0.58, 2.51)
Stroma-OS
Kojima 2005 ADC 129 2.04 (1.05, 3.96)
Welsh 2005 Mixed 162 1.20 (0.86, 1.68)
Subtotal (I-squared = 48.5%, p = 0.164) 1.34 (0.99, 1.81)
Stroma-DSS
Al-Shibli 2010 Mixed 335 1.10 (0.83, 1.45)
Hald 2013 Mixed 55 1.07 (0.94, 1.21)
Subtotal (I-squared = 0.0%, p = 0.864) 1.07 (0.96, 1.20)
.0702
1
14.2
B
NK cells
Study Year Histology N
HR (95% CI)
Tumour-DSS
Al-Shibli 2009 Mixed 335 1.70 (0.37, 7.73)
Hald 2013 Mixed 55 6.44 (0.39, 107.01)
Subtotal (I-squared = 0.0%, p = 0.414) 2.29 (0.61, 8.69)
Stroma-DSS
Al-Shibli 2009 Mixed 335 0.55 (0.34, 0.89)
Hald 2013 Mixed 55 0.21 (0.03, 1.33)
Subtotal (I-squared = 0.0%, p = 0.328) 0.51 (0.32, 0.82)
.00934
1
107

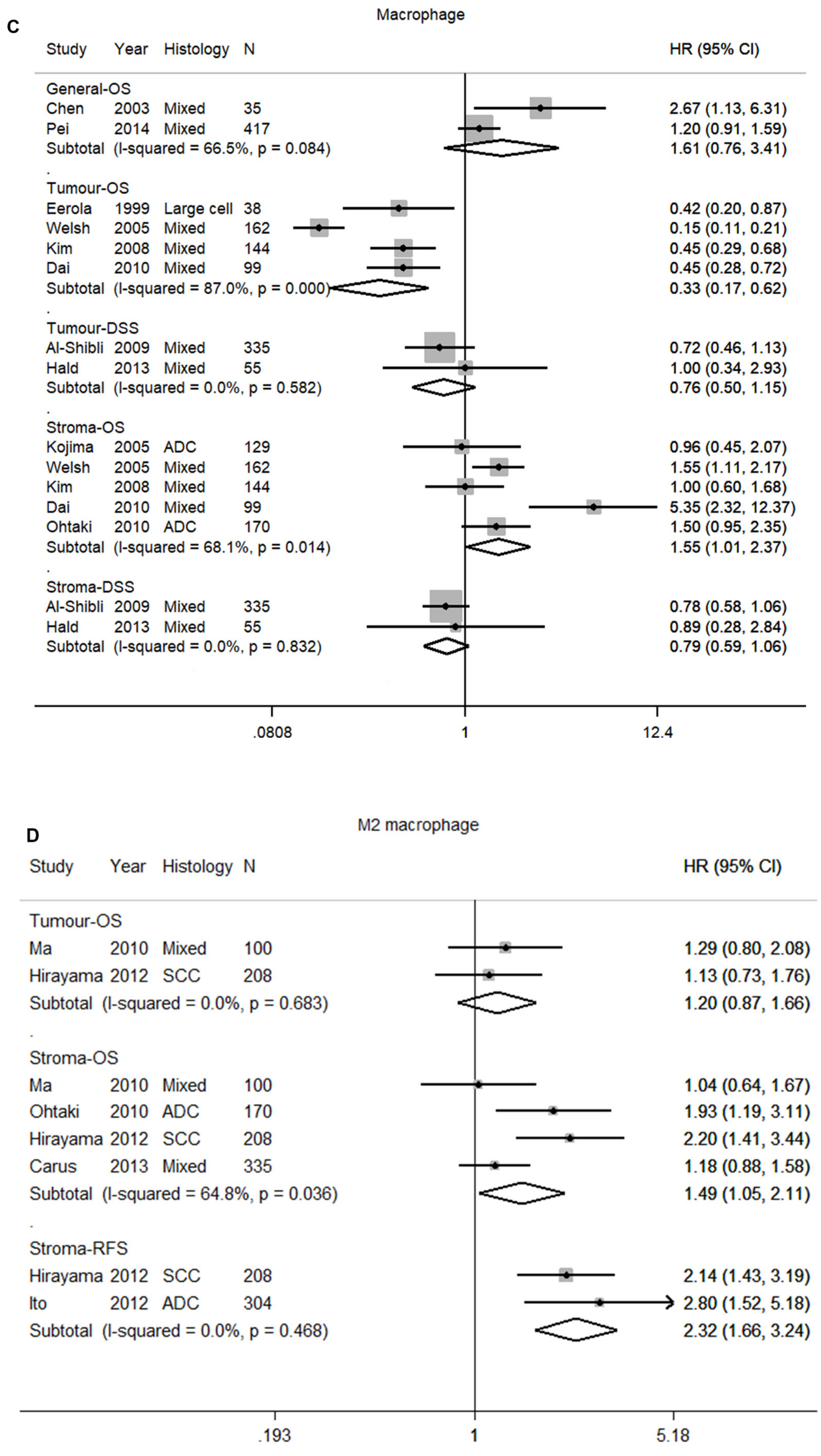
C
Macrophage
Study Year Histology N
HR (95% CI)
General-OS
Chen 2003 Mixed 35 2.67 (1.13, 6.31)
Pei 2014 Mixed 417 1.20 (0.91, 1.59)
Subtotal (I-squared = 66.5%, p = 0.084) 1.61 (0.76, 3.41)
Tumour-OS
Eerola 1999 Large cell 38 0.42 (0.20, 0.87)
Welsh 2005 Mixed 162 0.15 (0.11, 0.21)
Kim 2008 Mixed 144 0.45 (0.29, 0.68)
Dai 2010 Mixed 99 0.45 (0.28, 0.72)
Subtotal (I-squared = 87.0%, p = 0.000) 0.33 (0.17, 0.62)
Tumour-DSS
Al-Shibli 2009 Mixed 335 0.72 (0.46, 1.13)
Hald 2013 Mixed 55 1.00 (0.34, 2.93)
Subtotal (I-squared = 0.0%, p = 0.582) 0.76 (0.50, 1.15)
Stroma-OS
Kojima 2005 ADC 129 0.96 (0.45, 2.07)
Welsh 2005 Mixed 162 1.55 (1.11, 2.17)
Kim 2008 Mixed 144 1.00 (0.60, 1.68)
Dai 2010 Mixed 99 5.35 (2.32, 12.37)
Ohtaki 2010 ADC 170 1.50 (0.95, 2.35)
Subtotal (I-squared = 68.1%, p = 0.014) 1.55 (1.01, 2.37)
Stroma-DSS
Al-Shibli 2009 Mixed 335 0.78 (0.58, 1.06)
Hald 2013 Mixed 55 0.89 (0.28, 2.84)
Subtotal (I-squared = 0.0%, p = 0.832) 0.79 (0.59, 1.06)
.0808
1
12.4
D
M2 macrophage
Study Year Histology N
HR (95% CI)
Tumour-OS
Ma 2010 Mixed 100 1.29 (0.80, 2.08)
Hirayama 2012 SCC 208 1.13 (0.73, 1.76)
Subtotal (I-squared = 0.0%, p = 0.683) 1.20 (0.87, 1.66)
Stroma-OS
Ma 2010 Mixed 100 1.04 (0.64, 1.67)
Ohtaki 2010 ADC 170 1.93 (1.19, 3.11)
Hirayama 2012 SCC 208 2.20 (1.41, 3.44)
Carus 2013 Mixed 335 1.18 (0.88, 1.58)
Subtotal (I-squared = 64.8%, p = 0.036) 1.49 (1.05, 2.11)
Stroma-RFS
Hirayama 2012 SCC 208 2.14 (1.43, 3.19)
Ito 2012 ADC 304 2.80 (1.52, 5.18)
Subtotal (I-squared = 0.0%, p = 0.468) 2.32 (1.66, 3.24)
.193
1
5.18

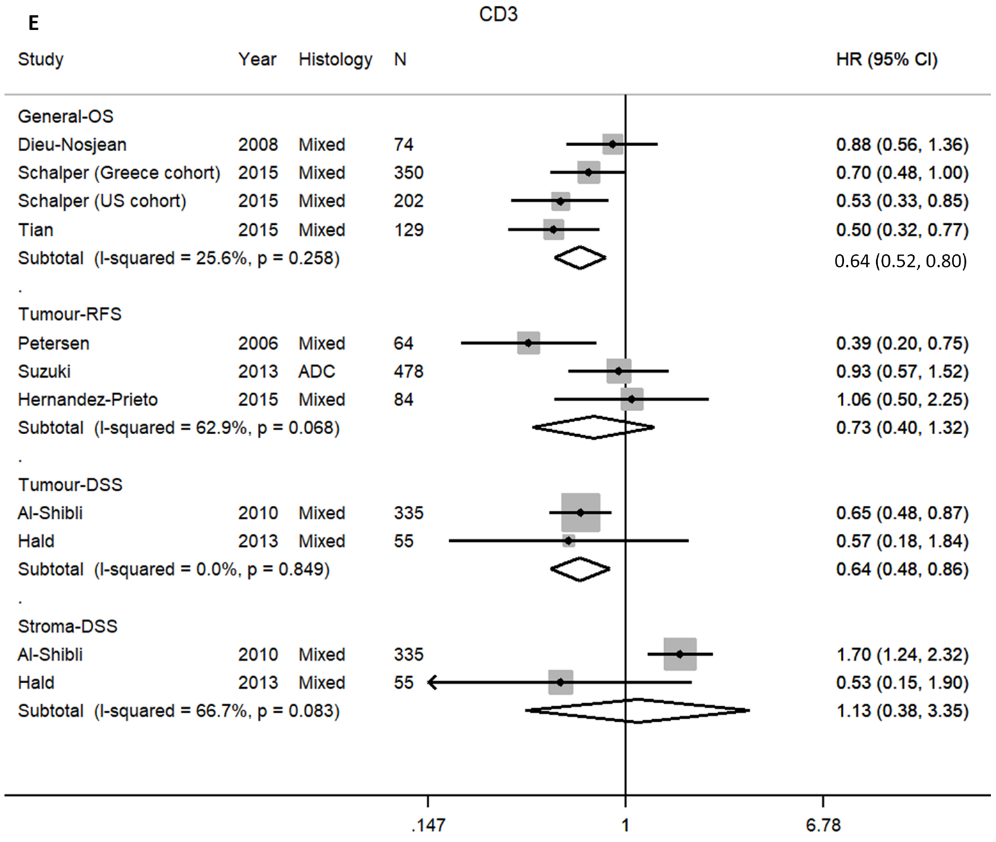
E
CD3
Study Year Histology N HR (95% CI)
General-OS
Dieu-Nosjean 2008 Mixed 74 0.88 (0.56, 1.36)
Schalper (Greece cohort) 2015 Mixed 350 0.70 (0.48, 1.00)
Schalper (US cohort) 2015 Mixed 202 0.53 (0.33, 0.85)
Tian 2015 Mixed 129 0.50 (0.32, 0.77)
Subtotal (I-squared = 25.6%, p = 0.258) 0.64 (0.52, 0.80)
Tumour-RFS
Petersen 2006 Mixed 64 0.39 (0.20, 0.75)
Suzuki 2013 ADC 478 0.93 (0.57, 1.52)
Hernandez-Prieto 2015 Mixed 84 1.06 (0.50, 2.25)
Subtotal (I-squared = 62.9%, p = 0.068) 0.73 (0.40, 1.32)
Tumour-DSS
Al-Shibli 2010 Mixed 335 0.65 (0.48, 0.87)
Hald 2013 Mixed 55 0.57 (0.18, 1.84)
Subtotal (I-squared = 0.0%, p = 0.849) 0.64 (0.48, 0.86)
Stroma-DSS
Al-Shibli 2010 Mixed 335 1.70 (1.24, 2.32)
Hald 2013 Mixed 55 0.53 (0.15, 1.90)
Subtotal (I-squared = 66.7%, p = 0.083) 1.13 (0.38, 3.35)
.147
1
6.78

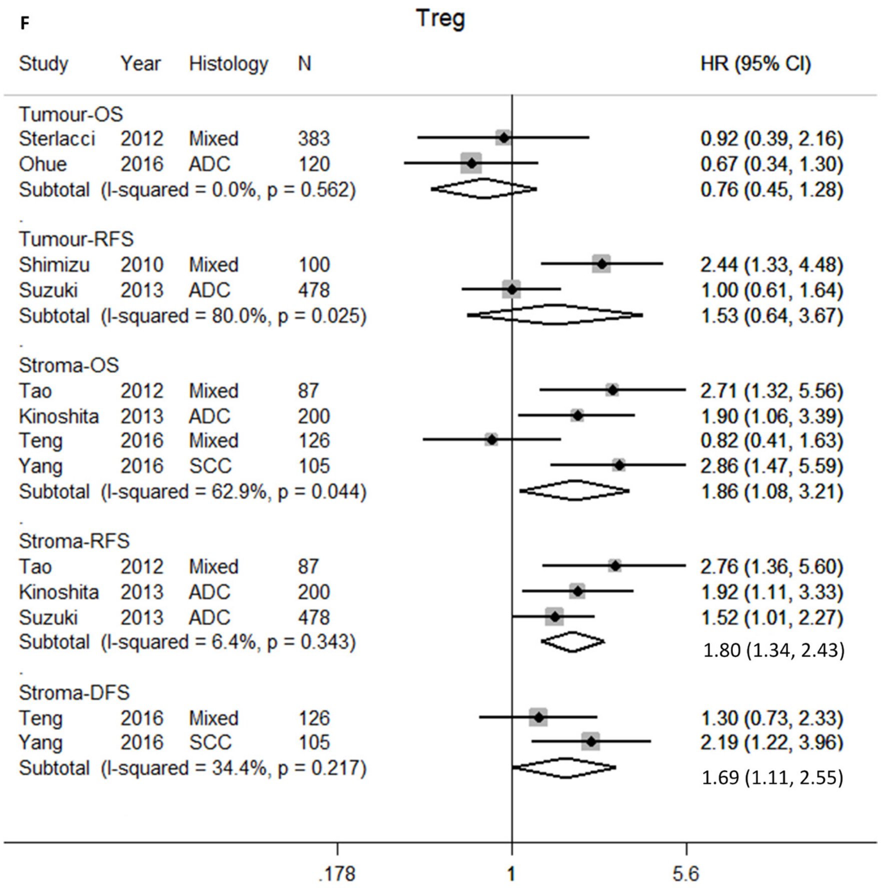
F
Treg
Study Year Histology N HR (95% CI)
Tumour-OS
Sterlacci 2012 Mixed 383 0.92 (0.39, 2.16)
Ohue 2016 ADC 120 0.67 (0.34, 1.30)
Subtotal (I-squared = 0.0%, p = 0.562) 0.76 (0.45, 1.28)
Tumour-RFS
Shimizu 2010 Mixed 100 2.44 (1.33, 4.48)
Suzuki 2013 ADC 478 1.00 (0.61, 1.64)
Subtotal (I-squared = 80.0%, p = 0.025) 1.53 (0.64, 3.67)
Stroma-OS
Tao 2012 Mixed 87 2.71 (1.32, 5.56)
Kinoshita 2013 ADC 200 1.90 (1.06, 3.39)
Teng 2016 Mixed 126 0.82 (0.41, 1.63)
Yang 2016 SCC 105 2.86 (1.47, 5.59)
Subtotal (I-squared = 62.9%, p = 0.044) 1.86 (1.08, 3.21)
Stroma-RFS
Tao 2012 Mixed 87 2.76 (1.36, 5.60)
Kinoshita 2013 ADC 200 1.92 (1.11, 3.33)
Suzuki 2013 ADC 478 1.52 (1.01, 2.27)
Subtotal (I-squared = 6.4%, p = 0.343) 1.80 (1.34, 2.43)
Stroma-DFS
Teng 2016 Mixed 126 1.30 (0.73, 2.33)
Yang 2016 SCC 105 2.19 (1.22, 3.96)
Subtotal (I-squared = 34.4%, p = 0.217) 1.69 (1.11, 2.55)
.178
1
5.6

G

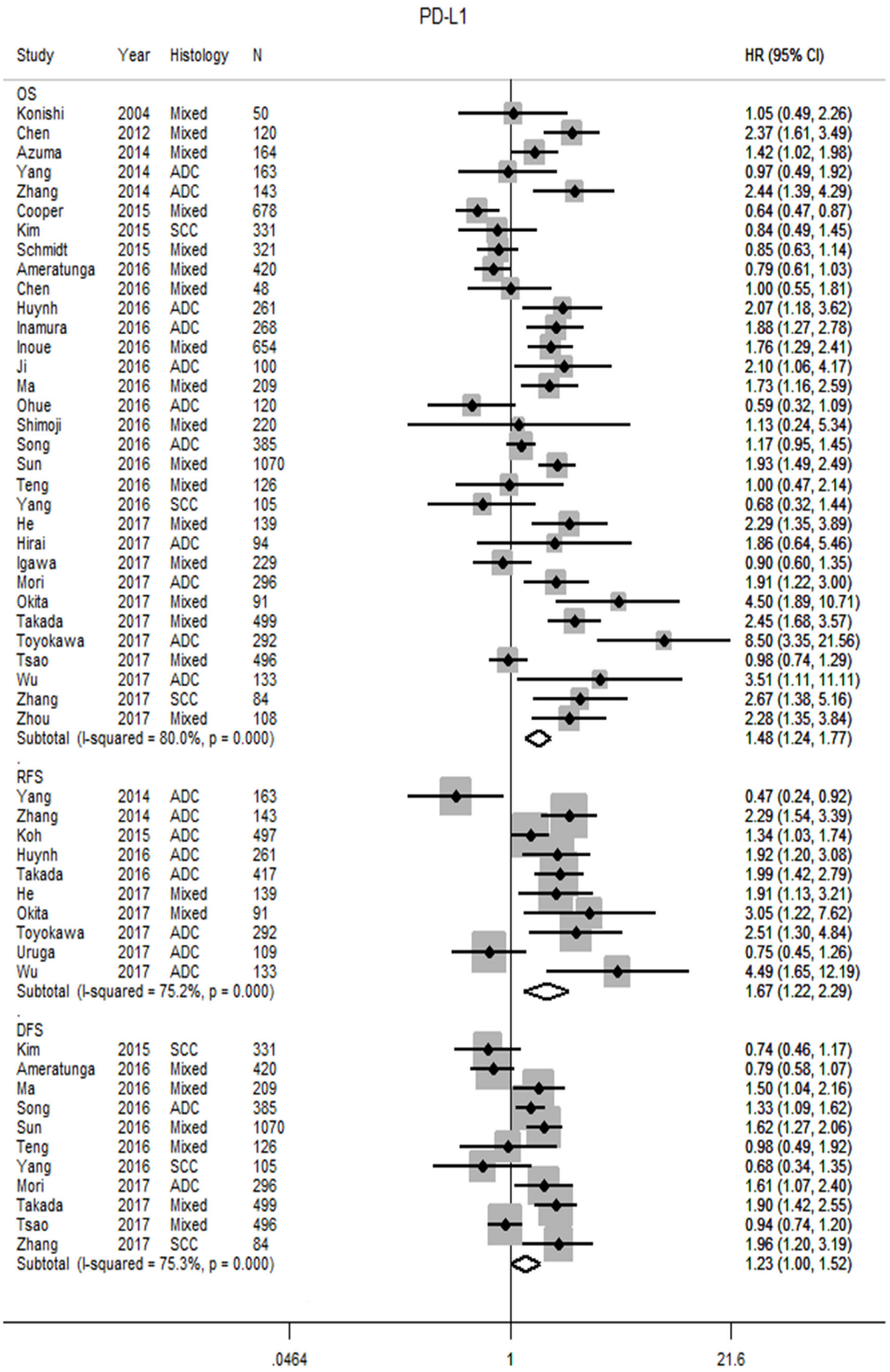

Supplementary Figure 3: Sensitivity analysis for performed by excluding studies of immune cells with quality score of three or less. Studies of (**A**) Mast cells, (**B**) NK cells, (**C**) macrophages, (**D**) M2 macrophages, (**E**) CD3+ T cells, (**F**) FOXP3+ T cells and (**G**) PD-L1 were re-analysed. Macrophages in general was not associated with overall survival and stromal FOXP3+ T cells was associated with poorer prognosis.

Supplementary Table 1: Study statistics. See Supplementary_Table_1

Supplementary Table 2: Summary of characteristics of studies included in meta-analysis. See Supplementary_Table_2

Supplementary Table 3: Stratification analysis according to ethnicity, publication year and sample size for the association between general location and OS for CD8 cells

**Stratification variable**	**No. of studies**	**No. of patients**	**Pooled HR (95% CI)**	***p*-value**	**Heterogeneity**	
					I^2	***p*-value**
Overall	7	1348	0.80 (0.56, 1.15)	0.232	87.2%	< 0.001
Ethnicity						
Asian	4	698	0.93 (0.43, 2.01)	0.849	91.6%	< 0.001
Caucasian	3	650	0.67 (0.45, 0.99)	0.048	79.0%	0.009
Publication year						
Before 2016	5	1110	0.88 (0.54, 1.43)	0.602	90.6%	< 0.001
After 2016	2	238	0.67 (0.50, 0.89)	0.007	0.0%	0.362
Sample size						
< 129	3	336	0.78 (0.60, 1.01)	0.061	53.0%	0.119
≥ 129	4	1012	0.87 (0.39, 1.96)	0.736	93.0%	< 0.001

Stratification based on cut-off point and histology resulted in only one study in the median, ADC and SCC stratum, thus analysis was not performed.

Supplementary Table 4: Stratification analysis according to ethnicity, publication year, sample size and cut-off point for the association between tumour and OS for CD8 cells

**Stratification variable**	**No. of studies**	**No. of patients**	**Pooled HR (95% CI)**	***p*-value**	**Heterogeneity**	
					I^2	***p*-value**
Overall	10	2916	0.78 (0.66, 0.93)	0.006	50.2%	0.034
Ethnicity						
Asian	5	466	0.83 (0.51, 1.36)	0.457	68.6%	0.013
Caucasian	5	2450	0.81 (0.72, 0.90)	0.001	24.9%	0.255
Publication year						
Before 2013	5	1914	0.89 (0.77, 1.02)	0.381	48.1%	0.103
After 2013	5	1002	0.73 (0.63, 0.84)	0.002	41.3%	0.146
Sample size						
< 113	5	444	0.81 (0.54, 1.21)	0.297	67.8%	0.014
≥ 113	5	2472	0.81 (0.71, 0.91)	0.003	28.9%	0.229
Cut-off point						
Median	3	552	0.72 (0.53, 0.99)	0.041	0.0%	0.557
Others	7	2364	0.80 (0.65, 0.99)	0.043	63.3%	0.012

Supplementary Table 5: Stratification analysis according to ethnicity, publication year, sample size and cut-off point for the association between stroma and OS for CD8 cells

Stratification variable	No. of studies	No. of patients	Pooled HR (95% CI)	*p*-value	Heterogeneity	
					I^2	*p*-value
Overall	8	2157	0.77 (0.69, 0.86)	< 0.001	9.3%	0.358
Ethnicity						
Asian	3	313	0.72 (0.51, 0.99)	0.050	0.0%	0.798
Caucasian	5	1844	0.77 (0.69, 0.87)	< 0.001	43.5%	0.132
Publication year						
Before 2015	4	648	0.67 (0.55, 0.81)	< 0.001	0.0%	0.834
After 2015	4	1509	0.81 (0.71, 0.93)	0.008	28.8%	0.239
Sample size						
< 107	4	366	0.83 (0.71, 0.97)	0.026	10.2%	0.342
≥ 107	4	1791	0.71 (0.61, 0.83)	< 0.001	0.0%	0.465
Cut-off point						
Median	2	163	0.67 (0.47, 0.97)	0.031	0.0%	0.376
Others	6	1994	0.78 (0.69, 0.87)	< 0.001	21.5%	0.272

Supplementary Table 6: Stratification analysis according to ethnicity, publication year, sample size and histology for the prognostic effect of PD-L1 on OS

Stratification variable	No. of studies	No. of patients	Pooled HR (95% CI)	*p*-value	Heterogeneity	
					I^2	*p*-value
Overall	35	8970	1.40 (1.18, 1.66)	< 0.001	80.8%	< 0.001
Ethnicity						
Asian	28	6350	1.63 (1.38, 1.93)	< 0.001	68.6%	< 0.001
Caucasian	7	2620	0.83 (0.66, 1.04)	0.101	66.2%	0.007
Publication year						
Before 2017	24	6509	1.18 (0.98, 1.43)	0.086	79.3%	< 0.001
After 2017	11	2461	2.19 (1.51, 3.16)	< 0.001	80.3%	< 0.001
Sample size						
< 164	17	1886	1.51 (1.14, 2.02)	0.005	72.6%	< 0.001
≥ 164	18	7084	1.32 (1.06, 1.64)	0.015	84.9%	< 0.001
Histology						
Mixed	21	6195	1.29 (1.03, 1.60)	0.025	83.7%	< 0.001
ADC	11	2255	1.79 (1.29, 2.48)	0.001	74.2%	< 0.001
SCC	3	520	1.15 (0.51, 2.60)	0.730	78.7%	0.009

Supplementary Table 7:. Stratification analysis according to ethnicity, publication year, sample size and histology for the prognostic effect of PD-L1 on DFS

Stratification variable	No. of studies	No. of patients	Pooled HR (95% CI)	*p*-value	Heterogeneity	
					I^2	*p*-value
Overall	12	4062	1.24 (1.01, 1.52)	0.040	72.9%	< 0.001
Ethnicity						
Asian	10	3146	1.38 (1.14, 1.67)	0.001	56.7%	0.014
Caucasian	2	916	0.88 (0.73, 1.06)	0.184	0.0%	0.386
Publication year						
Before 2017	8	2687	1.11 (0.86, 1.42)	0.427	69.2%	0.002
After 2017	4	1375	1.51 (1.01, 2.25)	0.044	81.8%	0.001
Sample size						
< 314	6	861	1.43 (1.16, 1.76)	0.024	35.2%	0.173
≥ 314	6	3201	1.17 (0.88, 1.55)	0.277	84.0%	< 0.001
Histology						
Mixed	7	2861	1.25 (0.94, 1.67)	0.132	78.2%	< 0.001
ADC	2	681	1.38 (1.15, 1.65)	< 0.001	0.0%	0.404
SCC	3	520	1.01 (0.51, 2.02)	0.975	80.1%	0.007

Supplementary Table 8: Stratification analysis according to ethnicity, publication year, sample size and histology for the prognostic effect of PD-L1 on RFS

Stratification variable	No. of studies	No. of patients	Pooled HR (95% CI)	*p*-value	Heterogeneity	
					I^2	*p*-value
Overall	10	2245	1.67 (1.22, 2.29)	0.002	75.2%	< 0.001
Ethnicity						
Asian	8	1875	1.82 (1.28, 2.57)	0.001	74.2%	< 0.001
Caucasian	2	370	1.21 (0.48, 3.03)	0.684	85.3%	0.009
Publication year						
Before 2017	5	1481	1.49 (1.01, 2.22)	0.047	80.0%	0.001
After 2017	5	764	2.02 (1.10, 3.74)	0.024	75.3%	0.003
Sample size						
< 153	5	615	1.98 (1.13, 3.44)	0.016	76.2%	0.002
≥ 153	5	1630	1.48 (0.97, 2.24)	0.067	78.2%	0.001
Histology						
Mixed	2	230	2.14 (1.36, 3.37)	0.001	0.0%	0.385
ADC	8	2015	1.56 (1.09, 2.25)	0.016	79.3%	< 0.001
